# Selective ferroptosis vulnerability due to familial Alzheimer’s disease presenilin mutations

**DOI:** 10.1038/s41418-022-01003-1

**Published:** 2022-04-21

**Authors:** Mark A. Greenough, Darius J. R. Lane, Rachelle Balez, Helena Targa Dias Anastacio, Zhiwen Zeng, Katherine Ganio, Christopher A. McDevitt, Karla Acevedo, Abdel Ali Belaidi, Jari Koistinaho, Lezanne Ooi, Scott Ayton, Ashley I. Bush

**Affiliations:** 1 Melbourne Dementia Research Centre, The Florey Institute of Neuroscience & Mental Health, The University of Melbourne, Parkville, VIC 3052 Australia; 2 Illawarra Health and Medical Research Institute, Northfields Avenue, Wollongong, NSW 2522 Australia; 3 School of Chemistry and Molecular Bioscience and Molecular Horizons, University of Wollongong, Northfields Avenue, Wollongong, NSW 2522 Australia; 4 Shenzhen Kangning Hospital & Shenzhen Mental Health Center, Shenzhen, China; 5 Department of Microbiology and Immunology, The Peter Doherty Institute for Infection and Immunity, The University of Melbourne, Melbourne, VIC 3000 Australia; 6 Neuroscience Center, University of Helsinki, Helsinki, Finland; 7 A.I. Virtanen Institute for Molecular Sciences, University of Eastern Finland, Kuopio, Finland

**Keywords:** Neural ageing, Neurological disorders

## Abstract

Mutations in presenilin 1 and 2 (*PS1* and *PS2*) cause autosomal dominant familial Alzheimer’s disease (FAD). Ferroptosis has been implicated as a mechanism of neurodegeneration in AD since neocortical iron burden predicts Alzheimer’s disease (AD) progression. We found that loss of the presenilins dramatically sensitizes multiple cell types to ferroptosis, but not apoptosis. FAD causal mutations of presenilins similarly sensitizes cells to ferroptosis. The presenilins promote the expression of GPX4, the selenoprotein checkpoint enzyme that blocks ferroptosis by quenching the membrane propagation of lethal hydroperoxyl radicals. Presenilin γ-secretase activity cleaves Notch-1 to signal LRP8 expression, which then controls GPX4 expression by regulating the supply of selenium into the cell since LRP8 is the uptake receptor for selenoprotein P. Selenium uptake is thus disrupted by presenilin FAD mutations, suppressing GPX4 expression. Therefore, presenilin mutations may promote neurodegeneration by derepressing ferroptosis, which has implications for disease-modifying therapeutics.

## Introduction

While Aβ plaques pathologically define Alzheimer’s disease (AD), a causal role for this peptide in instigating neurodegeneration remains contentious [1]. Familial AD (FAD) is an aggressive autosomal dominant disease caused by mutations of presenilin (PS) 1, PS2 or the amyloid-β (Aβ) precursor protein (APP), which are all involved in the generation of Aβ. PS is the catalytic subunit of γ-secretase, which cleaves APP to form Αβ [2]. Mutations often bias cleavage of APP to form longer, less soluble, 42- and 43-amino acid Aβ in preference to the more soluble 40 amino-acid peptide [3]. While the impact of PS mutations on increasing the ratio of Αβ42/40 has been highlighted as a signature of neurodegenerative toxicity [3], a systematic analysis of 138 familial PS mutations in vitro [4] revealed that the 42/40 ratio was not consistently elevated and, more tellingly, 90% of these mutations reduced activity, leading to *lower* production of Aβ 40 and 42, as observed in several other studies (e.g., [3–7]). This is consistent with a loss-of-function mechanism for disease pathogenesis, which is not surprising given the slim probability that more than 200 mutations scattered throughout the presenilin genes each causes disease by a common gain of function (i.e., increased Aβ42/40 ratio).

The basis of PS mutations conferring disease by causing a change in ratio of Aβ42/40 has therefore undergone renewed scrutiny. In addition to this “gain-of-toxic-function” model, it has been considered that PS mutations cause disease by loss-of-function. PS knockout is embryonically lethal by an uncertain mechanism [8]. Conditional inactivation of PS or another essential component of γ-secretase, nicastrin, caused neurodegeneration, inflammation and tau hyperphosphorylation in mice, despite lower Aβ production [9–11]. Knock-in of L435F or C410Y familial PS1 mutations that cause complete loss of both γ-secretase activity and Aβ generation [12, 13] caused neurodegeneration in mice similar to that of the conditional PS1/2 knockouts [14, 15]. A common mouse model, the APP/PS1 mouse, expresses the Swedish mutation of APP together with the ΔE9 truncated mutation of PS1 [16], resulting in cognitive impairment despite reduced γ-secretase activity [17]. Finally, a γ-secretase-inhibitor caused accelerated cognitive decline and other intolerable side effects that resulted in the early termination of clinical testing in over 3000 AD patients [18]. Collectively, these findings indicate that PS mutations might confer AD risk by *loss* of γ-secretase function.

In addition to APP, PS also cleaves >150 Type I transmembrane proteins [19], including Notch-1. Dysregulation of the Notch pathway has been considered a possible culprit in neuronal loss in PS-mutation familial AD [20]. Reduction of Notch signaling is a common feature of PS mutations [21–28]. Loss of Notch is embryonically lethal [29] and Notch receptors (1–4) are vital for cell survival during embryonic development [30]. γ-secretase cleaves Notch to release the Notch intracellular domain (NICD), which translocates to the nucleus and binds RBPJκ (**R**ecombination Signal **B**inding **P**rotein For Immunoglobulin *K*appa **J** Region) to form a transcription factor that regulates expression of genes involved in proliferation, differentiation, and cell survival [30]. Notch-1 is expressed by neurons in the adult brain and at particularly high levels in the hippocampus [31], which is affected early in AD. Reduced levels and activity of Notch-1 have been reported in hippocampal and cortical neurons isolated from sAD post-mortem brain tissue [32], consistent with reduced Notch-1 function in AD.

Brain iron accumulation with aging [33] is increasingly implicated in AD neurodegeneration [34, 35]. Brain iron levels, reflected by CSF ferritin [36–39], by Quantitative Susceptibility Mapping-MRI [40], or directly measured post-mortem [34, 35], predict cognitive deterioration in AD. Derepression of ferroptosis has been implicated as the mechanism underlying the association between brain iron burden in AD and neurodegeneration [41–47]. Ferroptosis is initiated by iron-mediated peroxidation of membrane polyunsaturated fatty acids into lethal lipid hydroperoxides, and cell death ensues when the checkpoint protection by the selenoprotein, glutathione peroxidase 4 (GPX4), is overwhelmed [48–50]. Peroxidation of membrane lipids is a normal and constant cellular event but does not proceed to cell disruption and paracrine propagation because of GPX4 [51]. GPX4 requires selenocysteine within the active site of the enzyme [52]. Physiological cell-selenium supply involves uptake of selenoprotein P (SELENOP), via transmembrane receptors, LRP2 and LRP8 [53]. In neurons, the main receptor is LRP8, whose gene is a newly described Notch target [54]. GPX4 consumes glutathione (GSH) for activity, and GSH depletion (as happens in the brain in AD [55–57]) suppresses GPX4 activity and enables ferroptosis to proceed [49, 50].

Ferroptosis is readily harnessed in cell culture, where it is induced by GSH depletion (e.g., by erastin) [58] or GPX4 inhibition (e.g., by RSL3) [49] and selectively rescued by potent radical trapping agents (e.g., ferrostatin and liproxstatin, which are not developed for human use) [59, 60]. Using these models, we investigated the impact of PSs on ferroptosis. Loss of PS function, by knockout or by pathogenic mutation, derepresses ferroptosis. Notch-1 and LRP8 emerge as critical mediators of ferroptosis through impacting GPX4 expression.

## Methods

### Cell culture

HEK293 cells (ATCC, Manassas, VA), HT22 cells (Sigma-Aldrich, Australia), or WT, PS KO and PS mutant MEFs (a kind gift of Prof. Bart De Strooper) were cultured in DMEM + GlutaMAX™-I supplemented with 10% (v/v) FBS (Bovogen, France), 1 mM sodium pyruvate (Thermo, Australia), and 100 U/ml penicillin-streptomycin (Thermo, Australia) at 37 °C, 5% CO_2_. All cell lines described above were routinely tested for mycoplasma (Cerberus Sciences, Australia).

All human iPSC experiments were conducted in accordance with the requirements of the University of Wollongong Human Ethics Research Committee (HE 13/299). The iPSCs used in this study were derived from dermal fibroblasts and have been previously described [61–65], with line details provided in (Extended Data Table 1). All iPSC cultures were maintained on Matrigel^TM^ (Corning) coated 60 mm tissue culture dishes (Greiner Bio-One, Austria) in mTeSR-1^TM^ medium (Stemcell Technologies, Canada) in a humidified incubator (Thermo, Australia) at 37 °C and 5% O_2_. The mTeSR-1^TM^ medium was changed daily and colonies were passaged every 5–7 days with Dulbecco’s phosphate-buffered saline (DPBS) + EDTA (Life Technologies, USA). All iPSC lines were regularly tested in-house for mycoplasma.

The healthy control, sporadic AD and PS1 A246E FAD iPSCs were differentiated into neurons using growth factors via neurospheres, as described previously [63]. Data from the healthy control lines and sporadic AD lines were combined by disease status for analysis.

### NGN2 lentiviral iPSC differentiation

The PS1ΔE9 FAD iPSCs and corresponding isogenic control were differentiated to neurons using NGN2 lentiviral transduction. Lentiviral particles were produced by co-transfection of the doxycycline-inducible lentiviral vector PLV-TetO-hNGN2-eGFP-PURO (NGN2; #79823, Addgene) or the reverse tetracycline transactivator vector FUW-M2rtTA (TTA; #20342, Addgene) by polyethyleneimine (Sigma-Aldrich, Australia) and Opti-MEM (Life-Technologies) transfection of HEK293T cells and packaged using plasmids vSVG (#8454, Addgene), RSV (#12253, Addgene) and pMDL (#12251, Addgene), following established methods [63].

The day prior to seeding iPSCs for differentiation (Day-3), 24 well plates (Greiner Bio-One, Austria) were coated with 10 μg/mL poly-d-lysine (Sigma-Aldrich, Australia) in DPBS for 30 min at room temperature. The poly-d-lysine was aspirated, and wells were washed 2× with DPBS before coating with 10 μg/mL laminin (Thermo, Australia) in DPBS overnight at 4 °C. Wells were washed 1× with DPBS before cells were seeded.

To seed iPSCs for differentiation (Day-2), cultures were single-cell passaged with DPBS + EDTA and accutase (Life Technologies, USA). Briefly, mTeSR-1^TM^ medium was aspirated, cultures washed with DPBS + EDTA and then incubated in fresh DPBS + EDTA until colonies started to fragment. The DPBS + EDTA was aspirated, and the cultures were then incubated with accutase until they became single cells. DMEM/F12 was then added, the cells aspirated and then centrifuged for 5 min at 300 × g. The DMEM/F12 was aspirated, the cells were gently resuspended in mTeSR-1^TM^ medium and cell concentration was determined with a hemocytometer. Cells were seeded at a density of 2.5–5.0 × 10^4^ in each well of a 24-well plate in mTeSR-1^TM^ medium supplemented with 10 μM Y-27632 (Focus Bioscience, Australia), then incubated overnight.

For lentiviral transduction (Day-1), the mTeSR-1^TM^ medium was aspirated and replaced with fresh mTeSR-1^TM^ medium supplemented with Y-27632 and containing the NGN2 and TTA lentiviruses at appropriate titer (1–2 μL/mL). Plates were incubated overnight.

For lentiviral induction (Day-0), 1 μg/mL doxycycline (Sigma-Aldrich, Australia) was added to neural induction media [Neurobasal medium (Thermo, Australia), 1× N-2 supplement (Thermo, Australia), 1× B-27 with vitamin A supplement (Thermo, Australia), 1× Insulin-transferrin-Selenium-A (Thermo, Australia), 1 × GlutaMAX^TM^ (Life Technologies, USA)] supplemented with 10 μM SB431524 (Stemcell Technologies, Canada) and 0.1 μM LDN193189 (Stemcell Technologies, Canada). The mTeSR-1^TM^ medium was aspirated, neural induction medium was added, and the plates incubated overnight.

Prior to puromycin selection (Day-1), plates were imaged using an Incucyte Zoom Imaging System (Essen Bioscience, USA) to confirm induction of lentiviral expression by doxycycline. The medium was aspirated and fresh neural induction medium containing doxycycline, SB431524, LDN193189 and 0.5 μg/mL puromycin (Sigma-Aldrich, Australia) was added. The cells were incubated overnight, with this feeding regime continued with daily media changes until Day-4.

To commence the transition from neural induction medium to neuronal medium (Day-4), 25% (v/v) neuronal medium [BrainPhys Neuronal Medium (Stemcell Technologies, Canada), 1× N-2 supplement, 1× B-27 with vitamin A supplement] was combined with 75% (v/v) neural induction medium and supplemented with doxycycline and 10 ng/mL BDNF (Miltenyi Biotech, Germany). The neural induction medium was aspirated, transition medium was added, and the plates were incubated overnight.

To continue neuronal medium transition (Day 5), 50% (v/v) neuronal medium was combined with 50% (v/v) neural induction medium and supplemented with doxycycline and BDNF. The media was aspirated, fresh transition medium was added, and the plates were incubated for 48 h.

For inhibition of non-neuronal cell growth (Day 7), 2.5 μM Cytosine beta-d-arabinofuranoside hydrochloride, crystalline (AraC; Sigma-Aldrich, Australia) was added to 75% (v/v) neuronal medium combined with 25% (v/v) neural induction medium, supplemented with doxycycline and BDNF. The media was aspirated, fresh transition medium was added, and the plates were incubated for 48 h.

To complete neuronal medium transition (Day 9), the medium was aspirated and 100% (v/v) neuronal medium, supplemented with BDNF, was added. The plates were incubated for 48 h, and this feeding regime continued with half media changes every 48 h until day 22. Representative images of Neurogenin-2 induced human neurons [66] are shown in Extended Data Fig. 7.

To harvest neurons for use in experiments, the neuronal medium was aspirated, the cells washed 2× with DPBS and then mechanically detached into fresh DPBS. The cells were centrifuged for 5 min at 300×g, the DPBS aspirated, and the dry cell pellet stored at −80 °C.

### Immunocytochemistry

For immunocytochemical preparations of iPSC-derived neurons, cells were fixed with 4% (w/v) paraformaldehyde in PBS for 7 min, permeabilized with 0.5% (v/v) Triton X-100 (Sigma-Aldrich, Australia) in PBS for 10 min and then blocked in 10% (v/v) goat serum (Life Technologies, USA) in PBS for 1 h, with all incubations at room temperature. MAP2 primary antibody (1:100; Covance, USA) was incubated overnight at 4 °C, followed by the secondary antibody (1:1000 Life Technologies, USA) with nuclear counter stain Hoechst 33342 (1:1000; Life Technologies, USA) for 1 h at room temperature, with primary and secondary antibodies made in blocking solution. All coverslips were washed three times with 1× PBS between each step. Coverslips were mounted in Prolong Gold Antifade Mountant (Life Technologies, USA) and imaged within 1–2 weeks of staining. Images were captured using a Leica TCS SP8 confocal microscope and acquired using Leica Application Suite - Advanced Fluorescence (LAS AF) 2.6.1–7314 software (Leica Microsystems, Germany) and representative images are shown in (Extended Data Fig. 8).

### Cell viability assays

For cell viability assays, cells were seeded at 10,000 cells/well in 96-well culture plates. The following day, ferroptosis was induced for 24 hours using culture media supplemented with erastin or RSL3 (Selleckchem, USA), or rescued by co-culture with these ferroptosis inducers plus liproxstatin-1 (Sigma-Aldrich, Australia) or seleno-l-cystine (Sigma Aldrich, Australia) at concentrations as specified. Cell viability was quantified using the MTT assay as previously described [67], except that formazan solubilization was performed in 100 μL DMSO after removal of all media.

### Lipid peroxidation assay

BODIPY^TM^ 581/591 undecanoic acid (C11) dye (BODIPY-C11; Thermo, Australia) was used as a probe to detect the level of lipid peroxidation. Oxidation of the polyunsaturated butadienyl portion of BODIPY-C11 shifts its emission peak from ~590 nm to ~510 nm which was detected by flow cytometry. The BODIPY-C11 sensor together with flow cytometry have been routinely used to measure the level lipid peroxidation [68, 69]. To detect lipid peroxidation following treatment with RSL3 (1 μM, 3 h), cells were treated with 0.125 μM BODIPY-C11 for an hour prior to harvesting with trypsin. Following centrifugation (800 × *g*, 5 min), cell pellets were washed twice with sorting buffer (1 × PBS, without Ca^2+^ or Mg^2+^, containing: 1 mM Na-EDTA, 25 mM HEPES (pH 7.0), 1% (v/v) FBS and 100 U/ml penicillin-streptomycin) and resuspended with ~200 μL of buffer containing DAPI to exclude dead cells from analysis. A CytoFLEX analyzer (Beckman Coulter) was used to detect a shift in fluorescence.

### Plasmid purification

3XFlagNICD1 was a gift from Raphael Kopan (Addgene plasmid # 20183; http://n2t.net/addgene:20183; RRID:Addgene_20183) [70]. Puro-iNotch1IC was a gift from Danwei Huangfu (Addgene plasmid # 75338; http://n2t.net/addgene:75338; RRID:Addgene_75338) [71]. EX-Z9294-M03 (human LRP8 expression construct) was purchased from GeneCopoeia (USA). Following transformation, plasmid DNA was extracted from host bacteria using a DNA Miniprep Kit (Qiagen, Australia) according to the manufacturer’s instructions and quantified by spectrophotometry using a NanoDrop (Thermo, Australia).

### CRISPR-Cas9 knockout

Knockout cells were generated using the ribonucleoprotein (RNP) transfection method. Briefly, MEFs or HT22 cells were transfected by Nucleofection (Nucleofector™ 2b Device; Amaxa) with RNP complexes comprising a 1:1 molar ratio of Alt-R® *S. pyogenes* HiFi Cas9 Nuclease V3 (Integrated DNA Technologies; IDT) and duplexes of Alt-R CRISPR–Cas9 crRNA and Alt-R CRISPR–Cas9 tracrRNA-ATTO^TM^ 550 (IDT). Gene-specific sequences of crRNAs used were: *Psen1* GUAGUCCACGGCGACAUUGUGUUUUAGAGCUAUGCU; *Psen2* CAUCUACACGCCCUUCACGGGUUUUAGAGCUAUGCU; *Lrp8* – CUGCUCGGACAACAGCGACGGUUUUAGAGCUAUGCU; and *Notch1* – GGUAUUCACGCCGUCCACACGUUUUAGAGCUAUGCU. 48 h following transfection, single-cell cloning was performed by the limiting dilution method in 96-well plates. Clones were selected in normal growth media additionally containing 400 nM liproxstatin and 800 nM α-tocopherol quinone to prevent loss of cells due to ferroptosis. 2-weeks later several clones of each KO were identified and expanded for further experiments. Control cells were transfected in parallel under identical conditions with RNPs constructed using negative control (NC) crRNA (murine; IDT). Surviving NC clones were pooled and used as the appropriate control line for the gene-specific knockout clones. Knockout was confirmed via western blotting with at least two different antibodies.

### Plasmid and siRNA transfections

Transfection of plasmid constructs was performed via endocytosis of cationic liposomes using FuGENE HD Transfection Reagent (Promega, USA) at a 3:1 ratio of FuGENE Transfection 6 reagent per μg plasmid DNA. In all other respects, transfection was performed according to the manufacturer’s specifications. Similarly, transfection of human *Notch1* Stealth siRNA or scrambled control siRNA (Thermo, Australia) was performed using Lipofectamine RNAiMAX reagent (Thermo, Australia) according to the manufacturer’s specifications. Selection of Puro-iNotch1IC stable transfectants in PS dKO MEFs was achieved by selection in media containing 5 µg/mL puromycin.

### Quantitative real-time PCR analyses of mRNA levels

RNA was extracted and converted to cDNA in one-step using a TaqMan™ Fast Advanced Cells-to-CT™ Kit (Thermo). Relative expression levels of mRNAs encoding the selected genes were measured according to the manufacturer’s protocol by RT-qPCR using specific TaqMan gene expression assays (Thermo, Australia), the following genes were analyzed on a ViiA^TM^ 7 Real-Time PCR System using MicroAmp Endura Plates with optical adhesive covers: *Lrp8* (Cat. #: 4331182; Assay ID: Mm00474030_m1), *Gpx4* (Cat. #: 4351372; Assay ID: Mm04411498_m1), *Notch1* (Cat. #: 4331182; Assay ID: Mm00627185_m1) and β*-actin* (Cat. #: 4448489; Assay ID: Mm01205647_g1). The expression levels of *Lrp8* were normalized to β*-actin* and calculated as fold-change vs WT by the 2^–∆∆Ct^ method.

### Cell lysis and protein extraction

For protein extraction, cells were disrupted with a cell scraper in a lysis buffer consisting of 50 mM HEPES-Na, pH 7.2, 0.5 M NaCl, 0.5% (v/v) NP-40, 0.05% LDS and 1×Pierce EDTA-free protease inhibitor cocktail (Thermo, Australia) in Milli-Q H_2_O and then vortexed. Supernatant was recovered following centrifugation at 16,000 × *g* for 10 min at 4 °C, and protein was quantified using the Pierce BCA protein assay kit (Thermo, Australia), according to manufacturer’s instructions. The protein fraction was precipitated in 4 volumes of acetone at −20 °C overnight and pelleted by centrifugation at 16,000 × *g* for 10 minutes. Supernatant was discarded, and the pellets air-dried and resuspended at a concentration of 1–2 μg/μL in sample buffer consisting of 1× Bolt™ LDS sample buffer (Thermo, Australia), 1 × Pierce protease inhibitor cocktail (Thermo, Australia), 1 × sample reducing agent (Thermo, Australia) in Milli-Q H_2_O.

### SDS-PAGE and western blotting

Protein extracts were separated by 1D gel electrophoresis using NuPAGE 4–12% (w/v) Bis-Tris midi gels (Invitrogen, USA), and transferred onto PVDF membranes using an iBlot 2 transfer system (Invitrogen, USA), according to the manufacturer’s specifications. Immunoblots were performed to detect FLAG tag (1:1000, D6W5B, Cell Signaling Technology, USA), LRP8/APOER2 (1:2000; ab108208, Abcam, UK), *β*-ACTIN (1:5000, AC-15, Sigma-Aldrich, Australia), GPX4 (1:1000, ab125066, Abcam, USA), Notch1 (1:1000, D1E11, Cell Signaling Technology, USA), PS1 (1:1000, D39D1, Cell Signaling Technology, USA), or PS2 (1:1000, D30G3, Cell Signaling Technology, USA), SELENOP (B-9, Mouse mAb; sc-376858, Santa Cruz Biotechnology, Inc., USA), cleaved caspase 3 (Asp175; 1:1000, 5A1E, Rabbit mAb 9664; Cell Signaling Technology, USA), or cleaved caspase 7 (Asp198; 1:1000, D6H1, Rabbit mAb 8438) followed by appropriate HRP-conjugated secondary antibodies (1:5000, Thermo, Australia). Chemiluminescence was detected using Pierce ECL (Thermo, Australia) and visualized using the LAS-3000 Imaging System (Fujifilm, Japan) or Odyssey® Fc Imaging System (LI-COR Biosciences, Lincoln, NE). Representative blots for all proteins (including *β*-actin) are shown. Original western blots for all relevant figures are shown in “Supplementary Material—Original Blots”.

### Elemental analysis/inductively coupled plasma mass spectroscopy

Cell pellets were digested in a minimal volume of concentrated nitric acid (Suprapur, Merck, Australia) overnight, followed by heating of the samples at 90 °C for 20 min to complete the digestion. The reduced volume after digestion (~20 μL) was diluted up to a final volume of 1 mL in Milli-Q water. Measurements of Se, Cu, Zn, Fe were made using an Agilent 8900 triple quadrupole inductively coupled plasma mass spectrometer (Agilent Technologies, Mulgrave, Australia) under operating conditions previously described [72]. The instrument was calibrated using blank, 10, 50, and 100 parts per billion (ppb) of a certified multi-element ICPMS standard solution (ICP-MS- CAl2–1, Accustandard, CT, USA). A certified internal standard solution containing 100 ppb of Yttrium via a T-piece was used as an internal control (ICP-MS- IS-MIX1-1, Accustandard, CT, USA). PBS and sample preparation blanks (containing Milli-Q water and nitric acid) were measured in each ICP-MS experiment to monitor for metal contamination. Data are expressed as μmol element/mg protein.

### Statistics

All statistical analyses were performed using GraphPad Prism® 8.0/9.0 (GraphPad Software, San Diego, CA). Dose–response data were modeled using non-linear curve fitting. Western blots were quantified by densitometric analyses using Fiji (ImageJ) software and Image Studio^TM^ Lite 5.0 (LI-COR Biosciences) and standardized to β-actin. Differences between two groups were determined using an unpaired two-tailed Student’s *t* test and were considered statistically significant when *P*  <  0.05, while differences between three or more groups were determined by one-way ANOVA with Bonferroni’s *post hoc* test of significance for multiple comparisons. The assumption of equal variances was validated by performing an F-test. Using the software package G*Power 3.1.9.7, the employed sample sizes were determined to provide adequate power (80–90%) to detect the relevant effect sizes (Cohen’s *d*) in each experiment. All experiments were replicated at least three times and results are expressed as mean ± S.D or S.E.M, as indicated in the figure legends; *,#*P*  <  0.05. **,##*P*  <  0.01. ***,###*P*  <  0.001. ****,####*P*  <  0.0001.

## Results

### Presenilins suppress ferroptosis

**Fig. 1:**
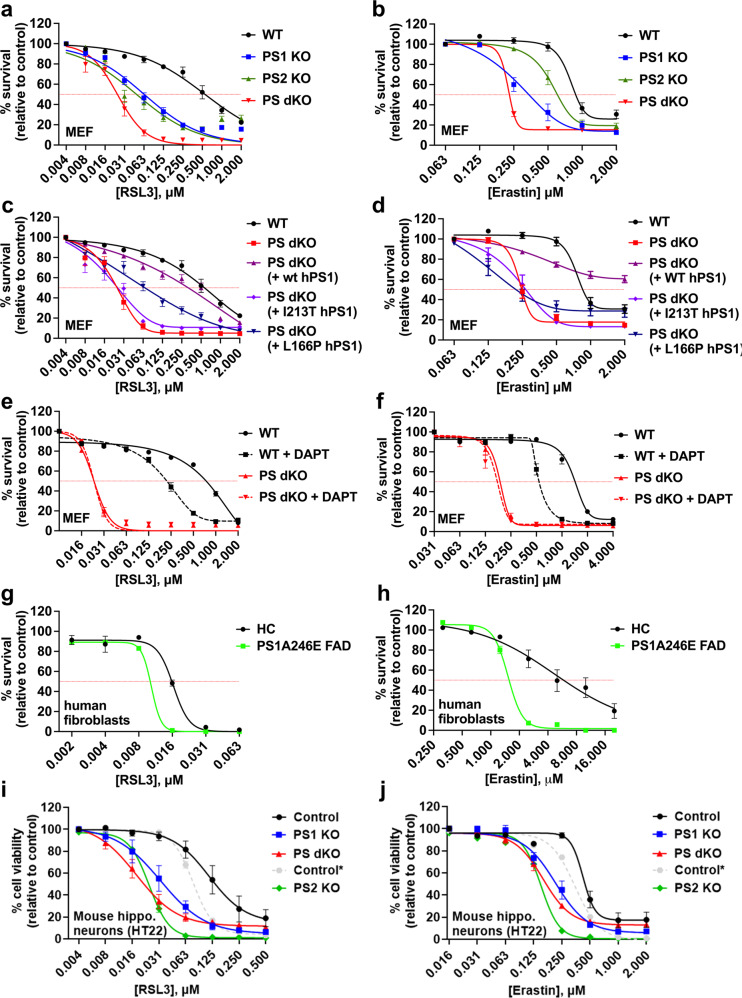
Presenilin confers resistance to ferroptosis in multiple cell types and is affected by familial Alzheimer’s disease causative mutations. Cell viability analyses performed using the MTT assay to compare ferroptosis resistance in mouse embryonic fibroblasts (MEFs), primary human fibroblasts and HT22 mouse hippocampal neurons to RSL3 (**a**, **c**, **e**, **g**, **i**) or erastin (**b**, **d**, **f**, **h**, **j**) at the concentrations shown, following 24 h incubation. Note: In **i** and **j**, “control” refers to matched negative-control HT22 cells transfected with non-targeting sgRNA contemporaneously with the generation of the PS1 KO and PS dKO HT22 cells, while “control*” is the contemporaneous and matched HT22 control for the subsequently created PS2 KO HT22 cells. Hence, PS1 KO and PS dKO HT22 should be compared to “control”, while PS2 KO should be compared to “control*”. Abbreviations: wildtype (WT), presenilin 1 knockout (PS1 KO), presenilin 2 knockout (PS2 KO), presenilin double-knockout (PS dKO), wild-type human sequence presenilin 1 (wt hPS1), L166P familial Alzheimer’s disease mutant human sequence presenilin 1 (L166P hPS1), I213T familial Alzheimer’s disease mutant human sequence presenilin 1 (I213T hPS1), healthy control human fibroblast (HC), A246E familial Alzheimer’s disease mutant presenilin 1 human fibroblast (PS1A246E FAD), N-[N-(3,5-Difluorophenacetyl)-L-alanyl]-S-phenylglycine t-butyl ester (DAPT, 10 μM, γ-secretase inhibitor). Data points represent mean percentage survival relative to respective controls ± SEM, *N* = 12–16 from 3 to 4 independent experiments.

Using cultured mouse embryonic fibroblasts (MEFs), human fibroblasts and mouse hippocampal neuronal-derived cells (HT22) we assessed cell viability after exposure to erastin or RSL3 to induce ferroptosis. In MEFs, loss of presenilin 1 (PS1 KO) or presenilin 2 (PS2 KO) conspicuously increased sensitivity to both RSL3- and erastin-induced ferroptosis, which was further exacerbated when both genes were ablated (PS dKO) (Fig. 1a, b). Lethality was rescued in all conditions by co-incubation with liproxstatin-1, an inhibitor of ferroptosis (Extended Data Fig. 1a, b). Ferroptosis is characterized by an accumulation of lethal lipid peroxides. Basal lipid peroxidation (assessed by the selective sensor, C11-BODIPY 581/591) was elevated in the PS dKO relative to WT cells under control conditions, which was significantly exacerbated in PS dKO cells compared to WT when challenged with RSL3 for 3 h, an incubation time that precedes overt cell death (Extended Data Fig. 1c). RSL3-induced lipid peroxidation was blocked by co-incubation with either seleno-l-cystine (SEC_2_) or liproxstatin-1 (Extended Data Fig. 1c), concomitant with rescue from lethality (Extended Data Fig. 1a).

We tested whether the enhanced susceptibility to ferroptosis in the PS dKO MEFs could be rescued by complementation with wild-type human or familial AD mutant PS1. Wild-type human PS1 (wt hPS1) was able to rescue the ferroptosis susceptibility phenotype to near WT MEF values, but PS dKO cells expressing either of two different familial AD mutant forms of PS1 (L166P and I213T) remained more sensitive to ferroptosis (Fig. 1c, d), consistent with impaired function.

To ascertain whether increased ferroptosis sensitivity in PS-deficient cells was due to loss of γ-secretase function, we pre-incubated WT and PS dKO MEFs for 72 h with the γ-secretase inhibitor DAPT prior to ferroptosis induction. Under these conditions, DAPT caused increased levels of the C-terminal fragments for APP and N-cadherin in WT, but not in PS dKO MEFs (Extended Data Fig. 2a, b), confirming the blockade of γ-secretase activity. As DAPT induced exaggerated sensitivity to both RSL3- and erastin- in WT but not PS dKO MEFs (Fig. 1e, f), these data are consistent with γ-secretase protecting against ferroptosis.

We studied the impact of endogenous FAD mutation using dermal fibroblasts derived from three related family members harboring the PS1A246E mutation. Indeed, fibroblasts from PS1A246E mutant carriers were significantly more susceptible to RSL3- or erastin-induced ferroptosis than age-matched healthy control fibroblasts (Fig. 1g, h) and in all cases were fully rescued with liproxstatin-1 (Extended Data Fig. 1d, e).

To assess whether disturbed PS function potentiates ferroptosis susceptibility in neuronal cells we used CRISPR-Cas9 technology to knockout PS1 (PS1 KO) or both PS1 and PS2 (PS dKO) in HT22 mouse hippocampal immortalized neuronal cells. An increased sensitivity to both RSL3 and erastin was observed in PS1, PS2, and PS1/PS2 KO HT22 cells compared to control cells transfected with non-specific sgRNA (Fig. 1i, j) and rescued by co-incubation with liproxstatin-1 (control, PS1 KO and PS dKO; Extended Data Fig. 1f, g), in concordance with the MEF and human fibroblasts results described above.

To determine if PS was merely protective against programmed cell death in general, we measured cell viability following treatment with well-characterized inducers of apoptosis. In contrast to the pronounced sensitization to ferroptosis by loss of PS (Fig. 1), neither PS dKO MEFs nor PS1 KO HT22 cells were more susceptible than WT controls to either staurosporine (Extended Data Fig. 3a, b) or camptothecin (Extended Data Fig. 3c, d). Interestingly, PS dKO MEFs were actually less sensitive to apoptosis (Extended Data Fig. 3a, c, e, fii, iii), which corroborates an earlier report by another group [73]. At the protein level, induction of ferroptosis was clearly distinguishable from the induction of apoptosis in WT and PS dKO MEFs by changes in levels of key proteins (Extended Data Fig. 3e, f). While no changes occurred in the levels of the apoptosis markers, cleaved caspases 3 and 7 (Extended Data Fig. 3e, fii, iii), erastin-induced a pathognomonic decrease in GPX4, which was most pronounced in WT cells that have high GPX4 relative to PS dKO cells (Extended Data Fig. 3e, fi). In contrast, camptothecin caused a pronounced increase in cleaved caspases 3 and 7 (Extended Data Fig. 3e, fii, iii), but no significant change in GPX4 (Extended Data Fig. 3e, fi). Thus, loss of PS sensitizes to ferroptosis but not apoptosis.

### Presenilins promote GPX4 expression

**Fig. 2:**
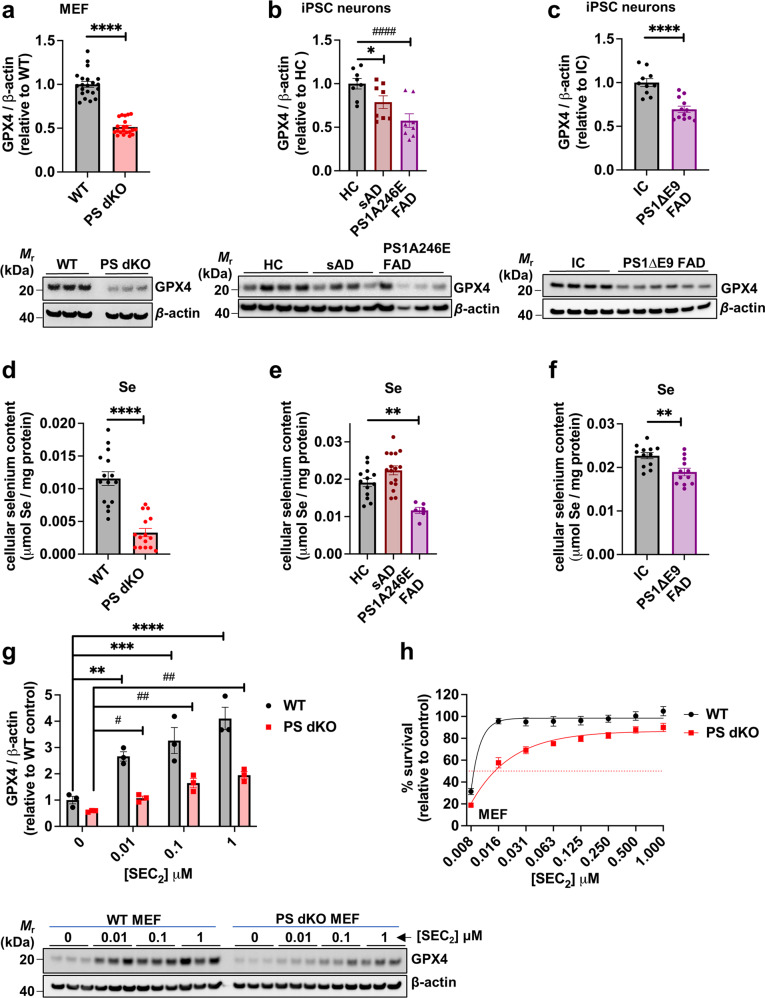
Presenilin impairment suppresses selenoenzyme glutathione peroxidase-4 (GPX4), the checkpoint for ferroptosis. Densitometric quantification and representative western immunoblots of whole cell lysates probed with specific antibodies raised against GPX4 or loading control β-actin are shown for; wildtype (WT) and presenilin double-knockout (PS dKO) MEFs (a); healthy control (HC), sporadic Alzheimer’s disease (sAD) and FAD presenilin 1 mutant (PS1A246E FAD) human iPSC-derived basal cholinergic neurons (b); CRISPR-corrected isogenic control (IC) and FAD presenilin 1 mutant (PS1ΔE9 FAD) human iPSC-derived neurons (**c**). Total cellular selenium content in: WT, PS1 KO, PS2 KO and PS dKO MEFs (**d**); HC, sAD or PS1A246E FAD mutant human iPSC-derived neurons (**e**); and human CRISPR-corrected isogenic control (IC) and corresponding PS1ΔE9 FAD iPSC-derived neurons (**f**). Western blot and densitometric quantification of whole cell lysates from WT or PS dKO MEFs probed with antibodies raised against GPX4 or β-actin, following pretreatment for 6 h with 0, 0.01, 0.1 or 1 μM seleno-l-cystine (SEC_2_) respectively (**g**). Cell viability of WT and PS dKO MEFs as assessed by MTT assay following co-treatment with 0.5 μM RSL3 and a dose range of SEC_2_, as shown (**h**). Western blot data represent mean values relative to their respective mean control value (±SEM) and individual values appear as points on each graph. For the western immunoblot and selenium content in iPSC basal cholinergic neurons each data point represents three pooled wells of a 24 well plate (see Extended Data Table 1 for pluripotent stem cell line details). For the cell viability assay, data are mean values (±SEM), *N* = 16.

To understand why PS-deficient cells are markedly susceptible to ferroptosis we examined GPX4, the selenoenzyme checkpoint for ferroptosis. GPX4 protein levels were ≈60% decreased in PS dKO MEFs compared to WT MEFs (Fig. 2a). This decrease in GPX4 was not due a decline in *Gpx4* transcript expression as *Gpx4* mRNA levels were unchanged (Extended Data Fig. 4). In induced basal cholinergic neurons derived from human iPSCs, we found that cells harboring the FAD point mutation PS1A246E, or the FAD deletion mutant PS1ΔE9, had conspicuous reductions in GPX4 compared to healthy age-matched controls (HC, Fig. 2b) or to isogenic CRISPR-corrected wild-type PS1 controls (Fig. 2c). Additionally, induced iPSC neurons from sporadic AD (sAD) patients expressed significantly less GPX4 than iPSC neurons from age-matched HC controls (Fig. 2b), indicating that a neuronal GPX4 lesion may be relevant to both familial and sporadic AD.

For its active site, GPX4 incorporates SEC, the 21st amino acid, via translational reprogramming involving its cognate tRNA (tRNA^Sec^) [74]. We found that PS dKO MEFs contain 71% less selenium than WT MEFs (Fig. 2d). Similarly, mutant PS1A246E neurons contained 39% less selenium and PS1ΔE9 iPSC neurons contained 17% less selenium than their respective controls (Fig. 2e, f), although selenium levels were not changed in neurons reprogrammed from cases of sporadic AD. Interestingly, despite a decrease in selenium in the FAD cells (Fig. 2e), there was no concomitant decrease in iron, copper and zinc levels (Extended Data Fig. 5d–f). However, in PS dKO MEFs there was a significant (*p* < 0.05) elevation in copper (31%; Extended Data Fig. 5b), and in PS1ΔE9 iPSC neurons we observed significant (*p* < 0.05) increases in both iron (26%) and zinc (23%; Extended Data Fig. 5g, i).

The reduced selenium levels in PS KO and mutant PS cells might account for their diminished GPX4 expression [52, 75]. Notably, GPX4 expression is also known to be decreased in a selenium-independent manner by ferroptotic stressors [76–79]. Considering this, the decrease of GPX4 (Fig. 2b) despite adequate selenium (Fig. 2e) in sAD induced neurons may reflect the presence of other sources of elevated ferroptotic stress relevant to AD (e.g., elevated redox-active iron, increased lipid peroxidation, decreased glutathione).

The main mechanism of selenium uptake in neurons is via the lipoprotein receptor LRP8, which binds to circulating SELENOP protein that serves as the principal selenium source (see below). Supplementing MEF cells with the organic selenium donor, SEC_2_, which bypasses the LRP8 uptake mechanism, induced a dose-dependent elevation of GPX4 in both WT and PS dKO MEFs (Fig. 2g), concomitantly suppressing lipid peroxidation (Extended Data Fig. 1c). Consistent with boosting GPX4 levels, SEC_2_ dose-dependently rescued cell viability following RSL3 intoxication of both WT and PS dKO cells (Fig. 2h).

### LRP8 suppression markedly sensitizes cells to ferroptosis

**Fig. 3:**
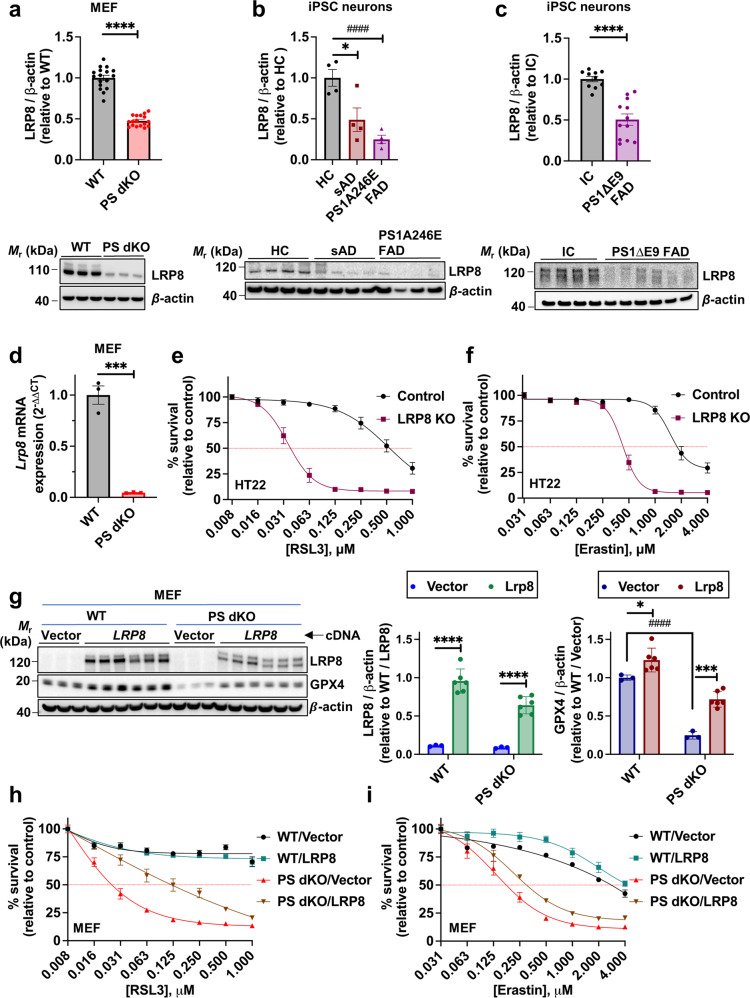
LRP8 mediates resilience against ferroptosis. Densitometric quantification and representative Western immunoblots of whole cell lysates probed with specific antibodies against LRP8 or loading control β-actin^ are shown for; wildtype (WT) and presenilin double-knockout (PS dKO) MEFs (**a**); healthy control (HC), sporadic Alzheimer’s disease (sAD) and FAD presenilin 1 mutant (PS1A246E FAD) human iPSC-derived basal cholinergic neurons (**b**); isogenic control (IC) and FAD presenilin 1 mutant (PS1ΔE9 FAD) human iPSC-derived basal cholinergic neurons (**c**) ^Note: Fig. 2b/3b and 2c/3c share the same β-actin blots as the displayed blots derive from the same original membranes that were successively probed with Lrp8, Gpx4 and β-actin antibodies (see “Supplemental Material – Original Blots”). *Lrp8* mRNA in WT and PS dKO MEFs as measured by RT-qPCR (**d**). MTT cell viability assay of LRP8 KO and control MEFs following 24 h exposure to a discriminating dose range of RSL3 (**e**) or erastin (**f**). Western immunoblot and quantification of LRP8 and GPX4 in WT and PS dKO MEFs that have been transiently transfected with human sequence *LRP8* or *Vector* cDNA (**g**). MTT cell viability assay of WT and PS dKO MEFs that have been transiently transfected with human sequence *LRP8* or *Vector* cDNA after 24 h exposure to a dose range of RSL3 (**h**) or erastin (i). Densitometric analyses of proteins relative to β-actin are shown as mean values (±SEM) and individual points represent independent wells of cultured cells except in the iPSC neurons which are pooled from three wells. For the cell viability assay, data are mean values (±SEM), *N* = 12.

LRP8 is known primarily as the cell surface receptor for Reelin, a large secreted neuronal glycoprotein with diverse functions in both the developing and adult brain, also implicated in the pathophysiology of AD [80]. LRP8 binding of SELENOP is the main pathway for selenium intake into neurons. Knockout of either *Lrp8* or *Selenop* in mice reduces brain selenium by more than 50% and causes severe neurodegeneration [81]. Several FAD PS1 mutations have been reported to impair LRP8 processing and trafficking [82]. We found that LRP8 protein expression in PS dKO MEFs was >50% suppressed compared to WT MEFs (Fig. 3a). Suppression of LRP8 and GPX4 levels by loss of PS in MEFs and HT22 cells (Extended Data Fig. 6a, bi, iii) occurred concomitantly with a decline in cellular SELENOP levels (Extended Data Fig. 6a, bii), consistent with the decrease in LRP8 lowering cellular SELENOP and selenium.

We also observed dramatic LRP8 loss in mature induced iPSC basal cholinergic neurons derived from pathogenic *PS1A246E* and *PS1ΔE9* mutation cases compared to controls (Fig. 3b, c). This reduction in protein was mirrored by a dramatic presenilin-dependent loss of *Lrp8* mRNA as measured by RT-qPCR (Fig. 3d), consistent with transcriptional regulation of *LRP8* being associated with presenilin. Thus, pathogenic presenilin mutations disturb LRP8-dependent selenium uptake into cells and consequently weaken the GPX4 ferroptosis checkpoint.

We tested the impact of loss of LRP8 on ferroptosis by CRISPR-Cas9 knock out of LRP8 in HT22 rat hippocampal neurons. The *Lrp8* knockout (*Lrp8* KO) HT22 cells were markedly more susceptible to ferroptosis induced by both RSL3 (Fig. 3e) or erastin (Fig. 3f). In a reciprocal experiment, transient transfection of human LRP8 in PS dKO MEFs, which are depleted of endogenous LRP8 (Fig. 3a, d), reversed the vulnerability to ferroptosis caused by the loss of PS (Fig. 3f). LRP8 overexpression induced GPX4 protein expression (Fig. 3g) and improved resilience against ferroptosis in PS dKO MEFs challenged with RSL3 (Fig. 3h) and, to a lesser extent, erastin (Fig. 3i).

### Notch-1 signaling regulates susceptibility to ferroptosis

Notch-1 is a substrate of presenilin-dependent γ-secretase processing, and its intramembrane cleavage releases the Notch intracellular domain (NICD) [83], which translocates to the nucleus where it regulates transcription. Reduced LRP8 expression has previously been reported in the hippocampi of Notch-1 knockout mice [84]. We reasoned that, if LRP8 is modulated at the mRNA level by presenilin (Fig. 3d), it could be dependent upon Notch signaling.

**Fig. 4:**
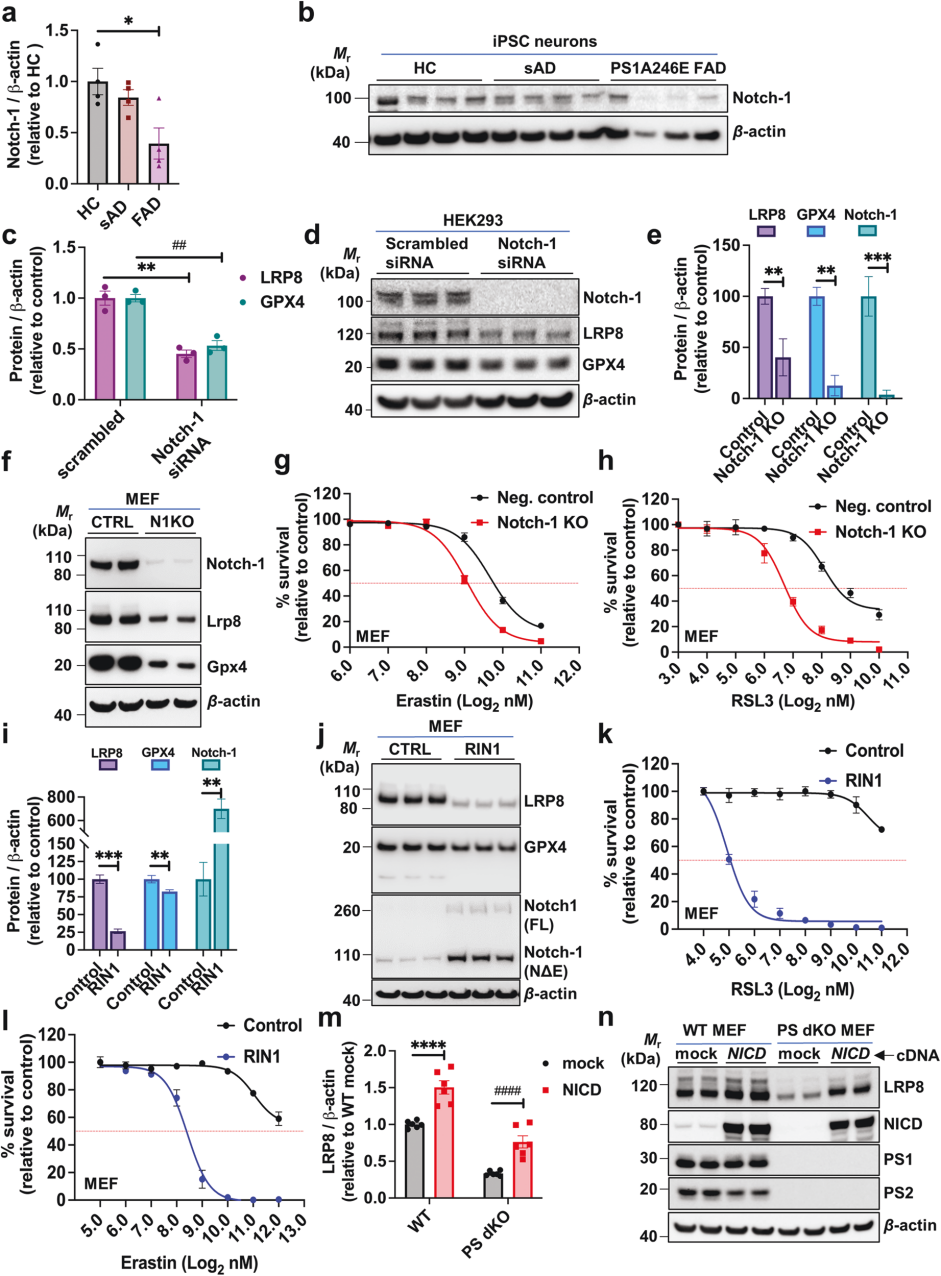
Notch-1 signaling regulates sensitivity to ferroptosis. Densitometric quantification of Notch-1 in whole cell lysates from human iPSC-derived basal cholinergic neurons (**a**) and corresponding western blot (**b**). Densitometric quantification of LRP8 and GPX4 in HEK293 cells following treatment with *Notch-1* specific siRNA or *scrambled* control siRNA (**c**) with the corresponding western blot shown in **d**. Densitometric quantification of LRP8, GPX4 and Notch-1 in Notch-1 CRISPR knockout (N1KO) and control (CTRL) MEF cell lysates (**e**) and a representative western blot shown in **f**. MTT cell viability assay of Notch-1 KO CRISPR knockout and CRISPR negative control MEFs following a 24 h incubation with a dose range of RSL3 (**g**) or erastin (**h**). Western blot of MEF cells incubated with RIN-1 or vehicle (CTRL) and probed with specific antibodies to LRP8, GPX4, Notch-1 and β-actin, as quantified in **i** with a representative western blot shown in **j**. MTT cell viability assays of MEF cells pre-incubated with RIN-1 (10 µM) or vehicle control (0.1% DMSO) for 48 h and following 24 h incubation with a dose range of RSL3 (**k**) or erastin (**l**). Quantification of LRP8 in WT and PS dKO MEFs 24 h post transfection with an NICD-containing plasmid or vector-only (mock) plasmid (**m**) and representative western blots demonstrating relative levels of LRP8, NICD, PS1, PS2 and β-actin (**n**). Densitometric analyses of proteins relative to β-actin are shown as mean values (±SEM) and individual points represent independent wells of cultured cells except in the iPSC neurons which are pooled from three wells. Abbreviations: HC = healthy control, sAD = sporadic Alzheimer’s disease, FAD PS1A246E = familial Alzheimer’s disease mutant presenilin 1 A246E, NICD = Notch intracellular domain, RIN-1 = RBPJ inhibitor-1; 2-(2-Fluorophenoxy)−4-(1-methyl-1H-pyrazol-5-yl) benzamide.

We noted that Notch-1 expression in human-derived iPSC neurons with the PS1A24E mutation was <50% lower relative to age-matched healthy control (HC) and sAD iPSC neurons (Fig. 4a, b). We investigated the impact of Notch-1 knockdown in human embryonic kidney (HEK293) cells (chosen because a high degree of transient knockdown is readily achievable and because they express measurable amounts of Notch-1, LRP8, GPX4, and γ-secretase component proteins). Notch-1 suppression by siRNA induced a ~50% reduction in LRP8 and GPX4 expression (Fig. 4c, d). Confirming the siRNA effects, CRISPR knockout of Notch-1 in MEFs caused a >50% reduction in LRP8 and GPX4 expression (Fig. 4e, f) and sensitized cells to RSL3-induced ferroptosis (Fig. 4g) and erastin-induced ferroptosis (Fig. 4h).

We hypothesized that NICD might regulate LRP8 expression via its function as a transcription factor upon binding within the RPBJ complex following its translocation to the cell nucleus. To investigate this, we blocked RPBJ transcriptional activity with RIN1 (**R**PBJ **IN**hibitor-1), a highly selective small molecule inhibitor [85]. Preincubation of WT MEF cells with RIN1 markedly decreased both LRP8 and GPX4 (Fig. 4i, j), consistent with disruption to Notch-1/NICD-dependent transcription of LRP8 and corresponding loss of GPX4. We surmised that loss of LRP8 leads to decresed selenium, as detected in PS dKO MEFs and FAD PS1 mutant iPSC neurons (as in Fig. 2d–f, respectively), and this loss of selenium decreases GPX4. Furthermore, impaired selenium uptake due to LRP8 depletion also suppresses the ability of the cell to synthesize more GPX4 to counteract lipid hydroperoxide stress. Indeed, inhibition of RPBJ in MEFs by RIN1 rendered the cells dramatically more sensitive to RSL3- and erastin-induced ferroptosis than vehicle-treated MEFs (Fig. 4k, l). To confirm that LRP8 expression was dependent on NICD, we transiently transfected NICD into MEFs. In reciprocal agreement with the Notch-1 siRNA, CRISPR knockout as well as the RIN-1 inhibitor results, overexpression of NICD promoted expression of LRP8 in MEFs (Fig. 4m, n). As stable expression of NICD in PS dKO MEFs restored *Lrp8* mRNA to WT levels (Extended Data Fig. 4), the NICD-dependent increase in LRP8 protein expression is likely due to increased message.

## Discussion

A growing body of evidence linking dysregulation of brain iron to AD progression [34–40] has implicated ferroptosis as a mechanism of neurodegeneration [86, 87]. In multiple cell types we demonstrated that genetic or pharmacological disruption of PS function sensitized cells to ferroptosis, but not apoptosis, which invites renewed interpretations for the role of PS in AD.

Familial PS mutations conferring loss of an essential or trophic function, such as ferroptosis suppression, is consistent with prior findings. For instance, conditional double knockout mice (cdKO) lacking both presenilin 1 and 2 in the postnatal mouse forebrain exhibit age-dependent neurodegeneration associated with hyperphosphorylated tau without the formation of Aβ plaques [88]. Furthermore, global expression of AD-causing *PS1* mutations mimics the neurodegeneration observed in the *PS1-*KO mouse [14]. Such findings support the well-evidenced alternative theory that FAD PS mutations cause clinical disease via pathologic sequelae of a loss of PS function (e.g., γ-secretase activity) in vivo [14]. Based on the findings presented here, we predict that one of these sequelae is an increased sensitization to ferroptosis downstream of diminished levels of neuronal selenium and GPX4. This perspective is consistent with previous findings in PS cdKO mice of increased levels of cortical neuronal cell death [10, 89], and with the dysregulation of genes associated with ferroptosis pathways (e.g., GPX5, ACSL1, SLC38A2 [9]). Consistent with our prediction, previous studies [90–92] have shown that therapeutic elevation of selenium in 3xTg-AD mice, which express mutant PS1 in addition to mutant APP and tau, effectively combats hippocampal-dependent learning and memory impairments, as well as behavioral phenotypes.

This perspective of loss-of-essential-PS-function in FAD differs from the amyloid cascade hypothesis, which postulates that the primary effect of PS mutations in FAD is through a toxic “gain-of-function”, by favoring the over-production of purportedly neurotoxic Aβ42 species [93]. While there are some exceptions [7], most studies report that PS mutations reduce Aβ42 levels even if the ratio to Aβ40 is often, but not always, increased [4–6, 94]. It is unclear how a reduction in Aβ42 production observed with many PS mutations could cause FAD and new perspectives are warranted.

**Fig. 5:**
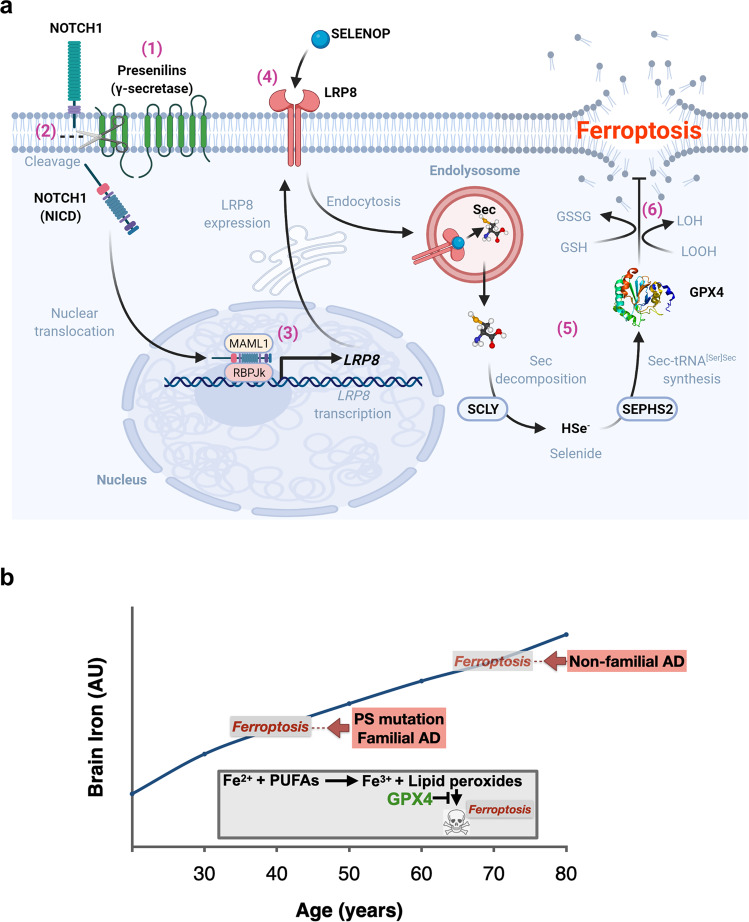
A hypothesis of how loss of γ-secretase function causes ferroptotic stress. **a (1)** Presenilins combine with other proteins of the γ-secretase complex to **(2)** cleave Notch-1, releasing the intracellular domain of Notch-1 (NICD). **(3)** NICD translocates to the nucleus and binds the co-activator, RBPJκ, to transcribe *LRP8*. **(4)** LRP8 is the neuronal receptor for the selenium transport protein, SELENOP. **(5)** Following internalization, SELENOP is degraded in the lysosome, releasing selenocysteine (Sec). Sec is decomposed to selenide by Sec lyase (SCLY), which is used to synthesize Sec-tRNA^[Ser]Sec^ in a pathway involving selenophosphate synthetase 2 (SEPHS2), ultimately incorporating Sec into *de novo* translated Glutathione Peroxidase 4 (GPX4). **(6)** GPX4, which detoxifies lethal iron-catalyzed lipid hydroperoxides (LOOH), is a critical checkpoint in the ferroptosis pathway. Diminished GPX4 activity causes ferroptosis. Created with BioRender.com. **b** Model for ferroptosis causing AD. Brain iron levels rise needlessly but universally with aging. The burden of brain iron increases the chance of ferroptosis occurring, held in better check by wild type PS than mutant PS. As a result, neuroferroptosis commences at an earlier age in people expressing mutant PS (familial AD) than the typical age of onset of non-familial AD in people expressing wild type PS. The insert shows the canonical ferroptosis events: cytoplasmic Fe^2+^ reacts with polyunsaturated fatty acids (PUFAs) to generate lipid peroxides, which, beyond a certain threshold, can disrupt the cell membrane. PS mutations suppress the expression of GPX4, which is a major checkpoint enzyme that prevent lipid peroxide accumulation from reaching lethal levels.

As mentioned above, dysregulated Notch signaling possibly contributes to neuronal loss in PS-linked familial AD [20]. For the first time, our findings indicate that Notch and LRP8 are critical for protecting against ferroptosis. As such, we propose that reduced Notch signaling (due to loss of PS function) contributes to neuronal dysfunction and death by ferroptosis in autosomal dominant PS-mutant carriers. Our findings indicate that both PS1 and PS2 regulate the LRP8-selenium-GPX4 axis via the Notch pathway (Fig. 5). The individual contributions of PS1 and PS2 to this machinery remain to be fully elaborated. In the absence of Notch, and thus the NICD produced by the γ-secretase, LRP8 expression is repressed [84], which we have here shown suppresses selenium and GPX4 levels, resulting in increased sensitivity to ferroptosis.

PS mutations commonly impair Notch processing [21], with some exceptions [94], but can also impair the trafficking of LRP8, which could also induce selenium deficiency in the absence of a Notch deficit [82]. Disruption to this axis could cause neurodegeneration by ferroptosis in AD, since neurodegeneration is a feature of mice lacking SELENOP or LRP8 [95], and of mice with conditional KO of GPX4 [96]. We demonstrate that decreased LRP8-mediated selenium uptake and GPX4 expression can be overcome by supplying non-SELENOP sources of selenium, such as SEC_2_ (Fig. 2g, h). Although SEC_2_-driven GPX4 expression was lower in PS dKO relative to WT cells (Fig. 2g), this is likely due to the lower basal selenium (Fig. 2d) and GPX4 (Fig. 2a, g) levels in PS dKO cells. Notably, the fold-increase of GPX4 from basal levels following SEC_2_ supplementation is similar between WT and PS dKO cells (Fig. 2g).

It is worth noting that in sAD induced neurons we observed a decrease in LRP8 (Fig. 3b), but no corresponding decline in selenium (Fig. 2e), which contrasts with the coupling of low LRP8 (Fig. 3b, c) and low selenium (Fig. 2e, f) in FAD PS-mutant induced neurons. The cell culture media of iPSC neurons contains some inorganic selenium (i.e., selenite) as an additive. While this was insufficient to rescue selenium in PS mutant induced neurons, which are severely deficient in selenium, it appeared sufficient to normalize selenium in non-PS mutant sAD cells. We hypothesize that in sAD induced neurons, unlike in PS mutant induced neurons, there is metabolic compensation for decreased LRP8 resulting in enhanced uptake of non-SELENOP-bound selenium.

Collectively, our studies demonstrate the potential for presenilin mutations to promote neurodegeneration irrespective of Aβ, which may or may not be an additional toxic lesion. Agents that rescue ferroptosis such as iron chelators, brain-accessible selenium treatments (not dependent on SELENOP), glutathione precursors and radical trapping agents may therefore have therapeutic potential for AD.

## Supplementary information

Extended Data Figure Legends and Extended Data Table 1

Extended Data Figure 1

Extended Data Figure 2

Extended Data Figure 3

Extended Data Figure 4

Extended Data Figure 5

Extended Data Figure 6

Extended Data Figure 7

Extended Data Figure 8

Supplemental Material - Original Blots

Reproducibility Checklist

## Data Availability

The data analysed during this study are included in this published article and the supplemental data files. Additional supporting data are available from the corresponding authors upon reasonable request.
