# Supplementary material for: Selective ferroptosis vulnerability due to familial Alzheimer’s disease presenilin mutations
Source: Cell Death Differ. 2022 Apr 21;29(11):2123–36. doi: 10.1038/s41418-022-01003-1 (PMC9613996; doi:10.1038/s41418-022-01003-1)
Supplement: Supplementary file 1 — Extended Data Figure Legends and Extended Data Table 1 [file 41418_2022_1003_MOESM1_ESM.docx]

**Extended Data Figure legends and Extended Data Table 1**

**Extended Data Fig. 1 Ferroptosis and associated lipid peroxidation is prevented by liproxstatin-1:** Cell viability analyses performed using the MTT assay to compare rescue of RSL3- or erastin-induced ferroptosis in MEFs **(a, b, h, i)**, human fibroblasts **(d, e)** and HT22 mouse hippocampal neurons **(f, g)** at the concentrations of inducers shown following a 24 h co-incubation with 1 μM liproxstatin-1 (**d-g**) or 20 µM of the pan-caspase inhibitor, Q-VD-OPh **(h, i)**. Relative levels of lipid peroxidation in WT and PS dKO MEFs measured using the ratiometric lipid peroxidation sensor BODIPY 581/591 C11 (Thermo, Australia) following a 3 h incubation in 1 μM RSL3 +/- 1 μM SEC_2_, +/- 1 μM liproxstatin-1 or BODIPY 581/591 C11 alone **(c)**. Fluorescence was measured by flow cytometry (see methods: lipid peroxidation assay) and data are expressed as a percentage of cells exhibiting a shift from red (peak ~590 nm) to green (~510 nm) associated with oxidation of the polyunsaturated butadienyl portion of the dye. DAPI-positive (i.e., dead) cells were excluded from the analysis. Data are mean values (+/- SEM), N = 12 from three independent experiments.

**Extended Data Fig. 2 Inhibition of gamma secretase activity by DAPT and loss or mutation of PS1:** Western blot analysis showing protein levels of PS1 CTF **(a, b*i*)**, APP CTF **(a, b*ii*)**, and N-cadherin (N-CAD) CTF **(a, b*iii*)**, relative to β-actin, in WT, PS dKO, PS dKO (+wt hPS1), PS dKO (+L166P hPS1) and PS dKO (+I213T hPS1) MEFs treated with/without DAPT (10 µM). Data are mean values (+/- SD, N = 2-6) from two **(b*i*)** or four **(b*ii,iii*)** independent experiments.

**Extended Data Fig. 3 Presenilin knockout does not sensitize cells to apoptosis:** MTT cell viability assay following 24 h incubation with the protein kinase inhibitor staurosporine in MEFs **(a)** and HT22 **(b)** or camptothecin, a topoisomerase 1 inhibitor, in MEFs **(c)** and HT22 **(d)**. Both staurosporine and camptothecin induce cell death via apoptosis. **(e, f*i-iii*)** Western blot analysis showing effects of a 12 h incubation with erastin (1 µM) or camptothecin (2 µM) on protein levels of GPX4 **(e, f*i*)**, cleaved caspase 3 **(e, f*ii*)** and cleaved caspase 7 **(e, f*iii*)**. Data are mean values (+/- SEM), N = 9 **(a-d)** or N = 3 **(e, f*i-iii*)** from three independent experiments.

**Extended Data Fig. 4 Changes in mRNA levels of *Lrp8*, *Gpx4* and *Notch1* in WT and PS dKO MEFs, and rescue of *Lrp8* by stable transfection with *NICD1*:** Relative mRNA levels of *Lrp8, Gpx4* and *Notch1* in WT, PS dKO and PS dKO (+*NICD1*) MEFs as measured by RT-qPCR. Data are mean values (+/- SD) from three independent experiments.

**Extended Data Fig. 5 Iron, copper and zinc content in cells:** Inductively coupled plasma mass spectroscopic measurement of total cellular iron, copper and zinc in MEFs **(a-c)** or iPSC induced basal cholinergic neurons **(d-i)**. Data are mean values (+/- SEM) and individual values are shown. Abbreviations: HC = healthy control; sAD = sporadic Alzheimer’s disease; FAD = familial Alzheimer’s disease; IC = isogenic control.

**Extended Data Fig. 6 Effects of loss of PS on protein levels of LRP8, SELENOP and GPX4 in MEFs and HT22 cells:** **(a, b*i-iii*)** Western blot analysis showing protein levels of LRP8 **(a, b*i*)**, SELENOP **(a, b*ii*)** and GPX4 **(a, b*iii*)**, relative to β-actin, in WT and PS dKO MEFs and HT22 cells. Data are mean values (+/- SD, N = 3) from three independent experiments.

**Extended Data Fig. 7 Neurogenin 2 derivation of human iPSC neurons:** Representative phase contrast and Neurogenin 2- GFP fluorescence microscopic images to depict neuronal morphology of PS1ΔE9 iPSC-derived basal cholinergic neurons **(a, b)** and the corresponding CRISPR-corrected control cells **(c, d)**.

**Extended Data Fig. 8 Expression of Microtubule-associated protein 2 (MAP2), a marker of neuronal differentiation:** Representative confocal microscopic images of MAP2 expression in neurons from (HC) healthy control **(a)**, sAD **(b)** and PS1A246E FAD **(c)** differentiated iPSC cell lines. MAP2 (red) was detected with a human anti-MAP2 antibody and cell nuclei were counter stained with Hoechst 33342 (blue).

**Extended Data Table. 1 Induced pluripotent stem cell line details:**

| **iPSC line** | **Sex** | **Age** | **Mutation** | ***ApoE* genotype** |
| --- | --- | --- | --- | --- |
| **HC 1** | Female | 56 | N.A. | ε2/4 |
| **HC 2** | Male | 57 | N.A. | ε3/3 |
| **HC 3** | Female | 75 | N.A. | ε2/3 |
| **SAD 1** | Female | 83 | N.A. | ε4/4 |
| **SAD 2** | Male | 65 | N.A. | ε3/4 |
| **SAD 3** | Male | 83 | N.A. | ε4/4 |
| **FAD** | Female | 56 | *PS1*A246E | ε3/4 |
| **FAD** | Female | 47 | *PS1*ΔE9 | ε3/3 |
| **IC** | Female | 47 | *PS1*ΔE9 corrected | ε3/3 |

Abbreviations: HC = healthy control; SAD = sporadic Alzheimer’s disease; FAD = familial Alzheimer’s disease; IC = isogenic control.
