# Supplementary figures and images for: Selective ferroptosis vulnerability due to familial Alzheimer’s disease presenilin mutations
Source: Cell Death Differ. 2022 Apr 21;29(11):2123–36. doi: 10.1038/s41418-022-01003-1 (PMC9613996; doi:10.1038/s41418-022-01003-1)

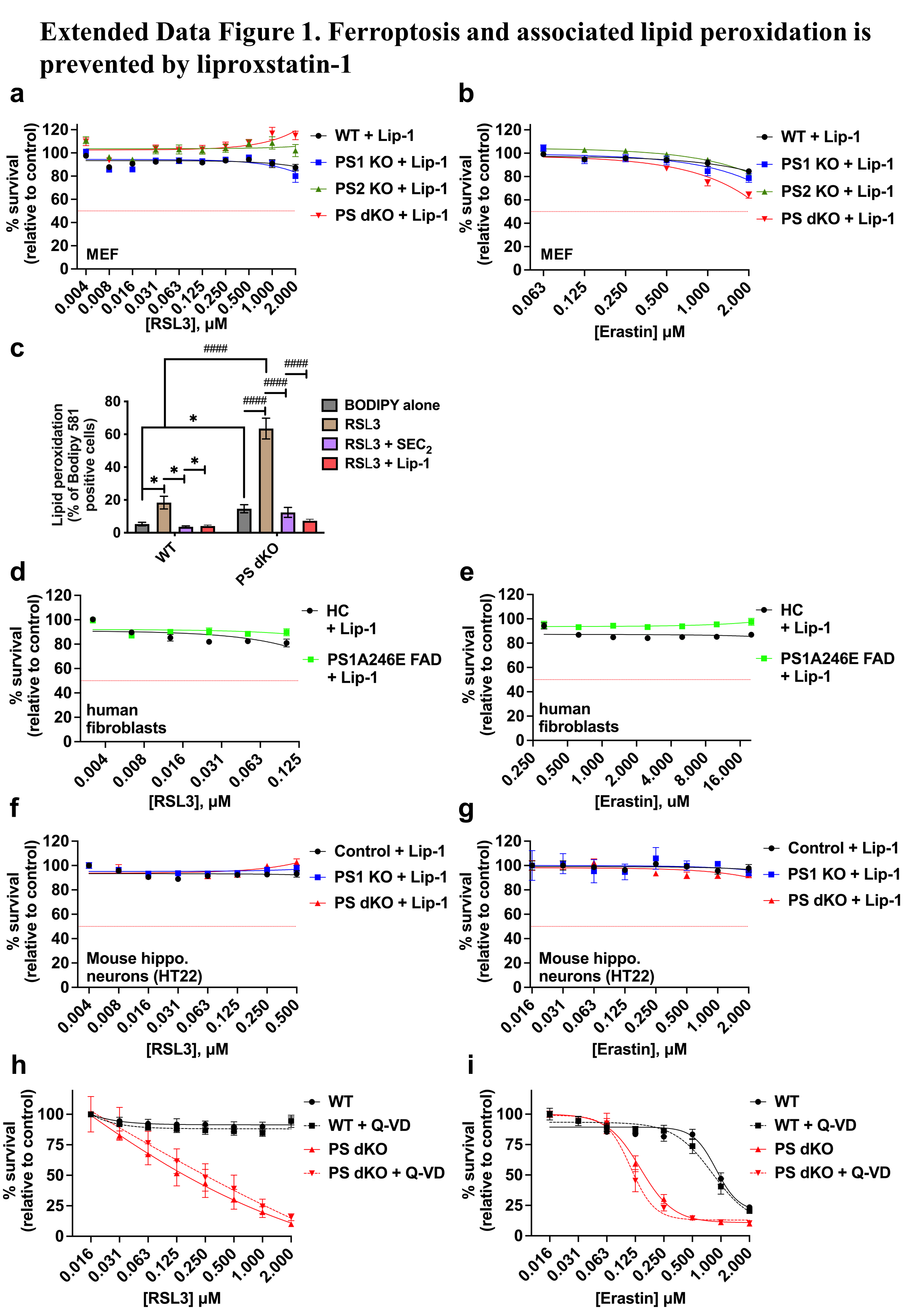

Supplement: Supplementary file 2 — Extended Data Figure 1 [file 41418_2022_1003_MOESM2_ESM.png]

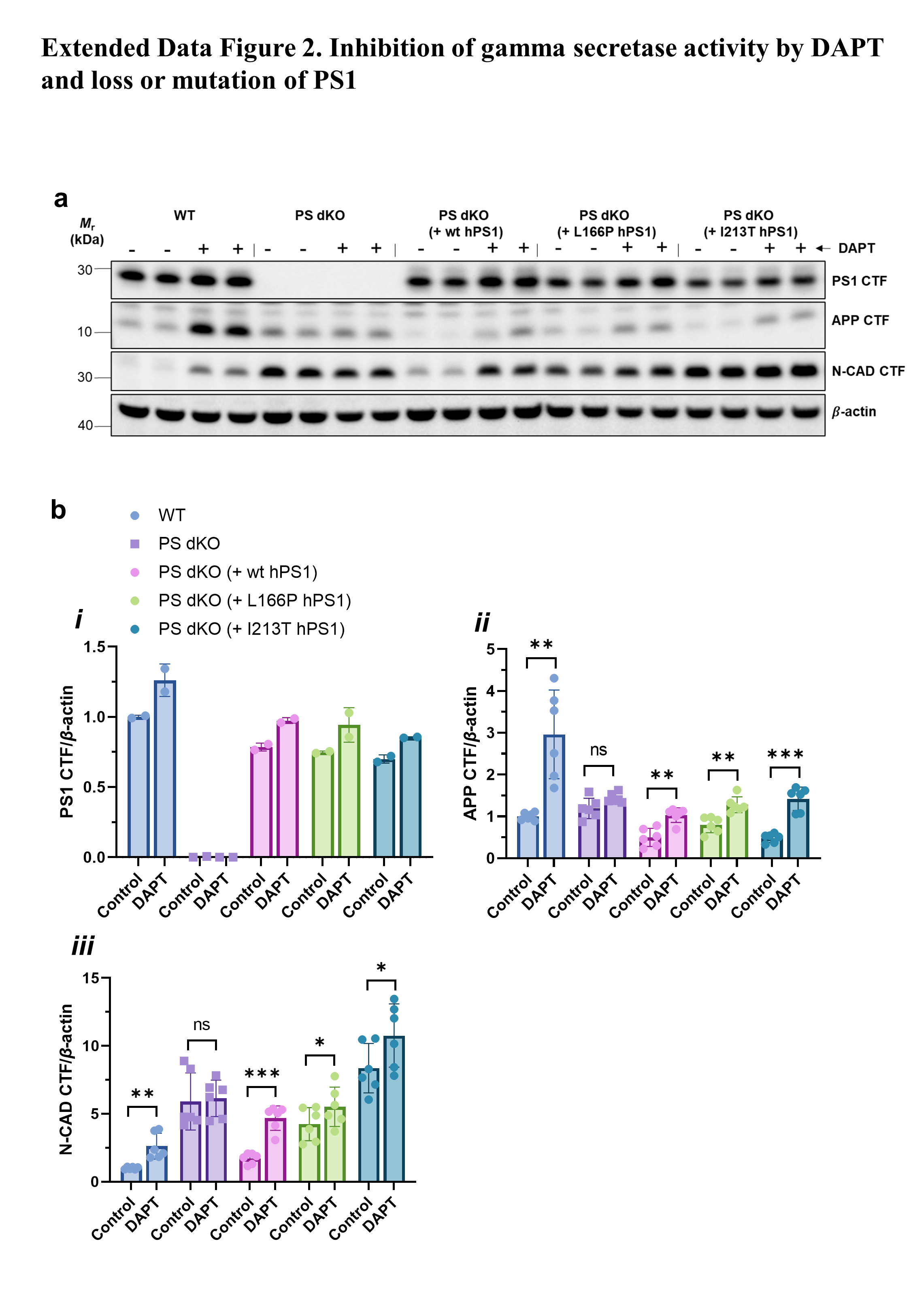

Supplement: Supplementary file 3 — Extended Data Figure 2 [file 41418_2022_1003_MOESM3_ESM.png]

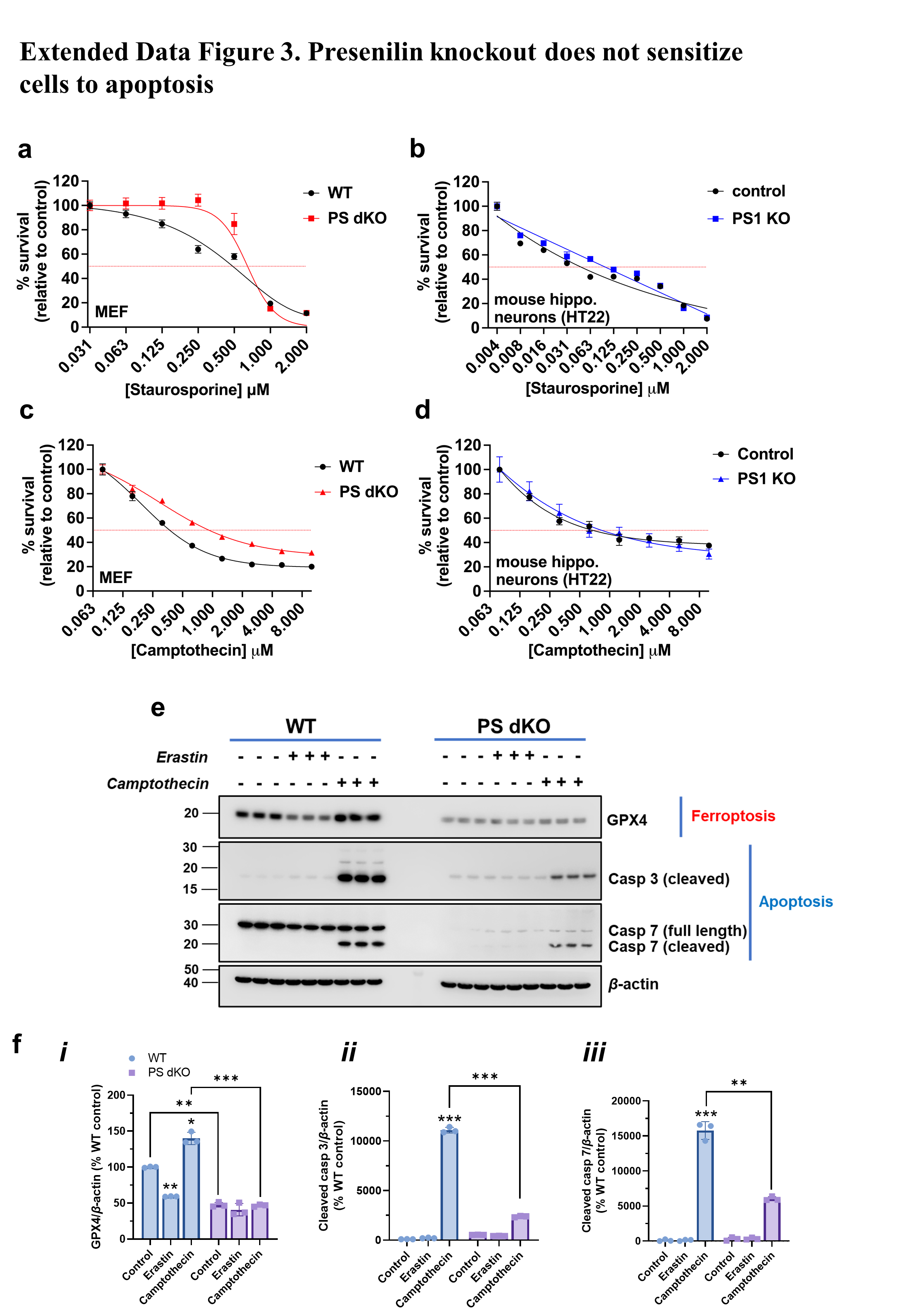

Supplement: Supplementary file 4 — Extended Data Figure 3 [file 41418_2022_1003_MOESM4_ESM.png]

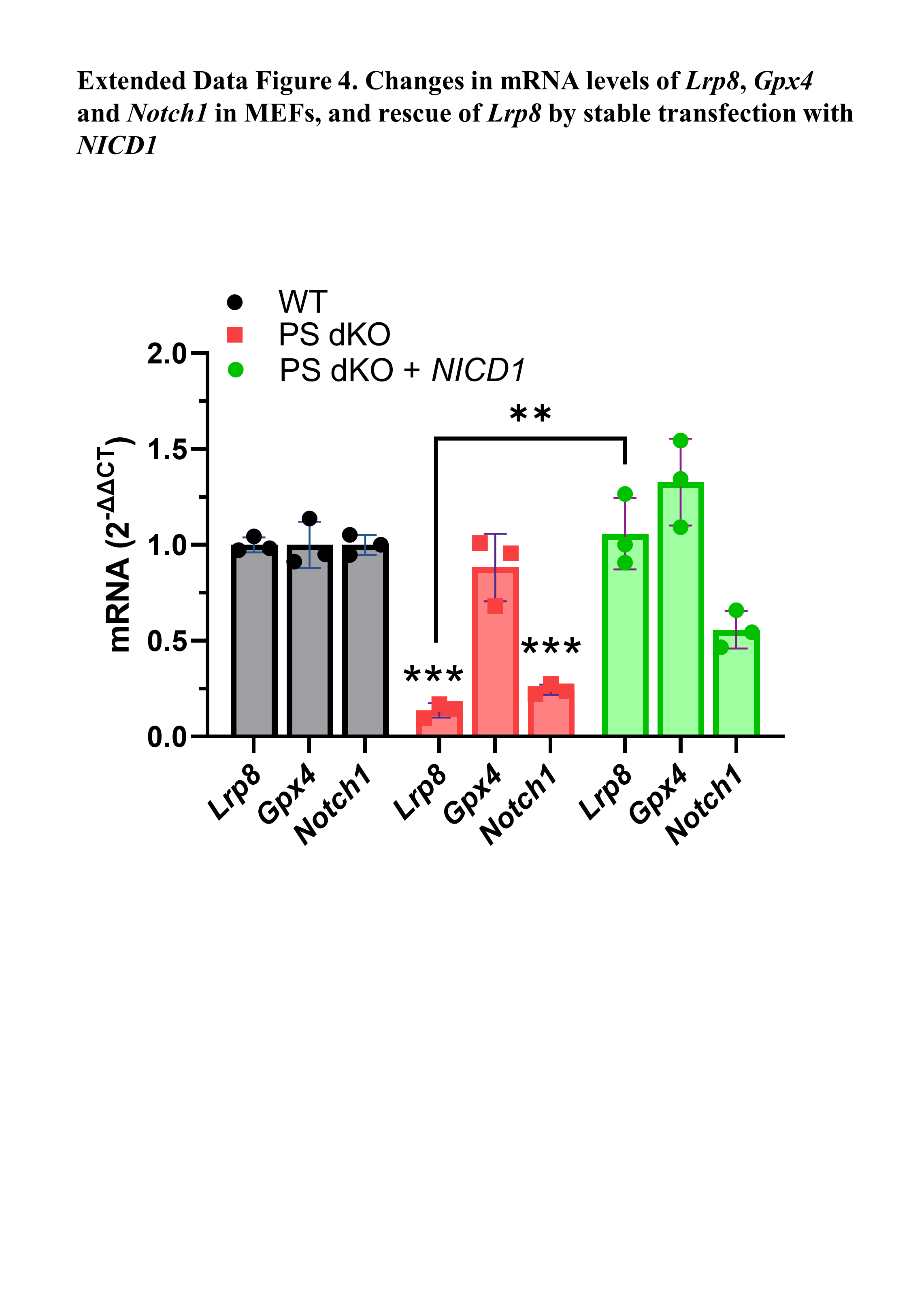

Supplement: Supplementary file 5 — Extended Data Figure 4 [file 41418_2022_1003_MOESM5_ESM.png]

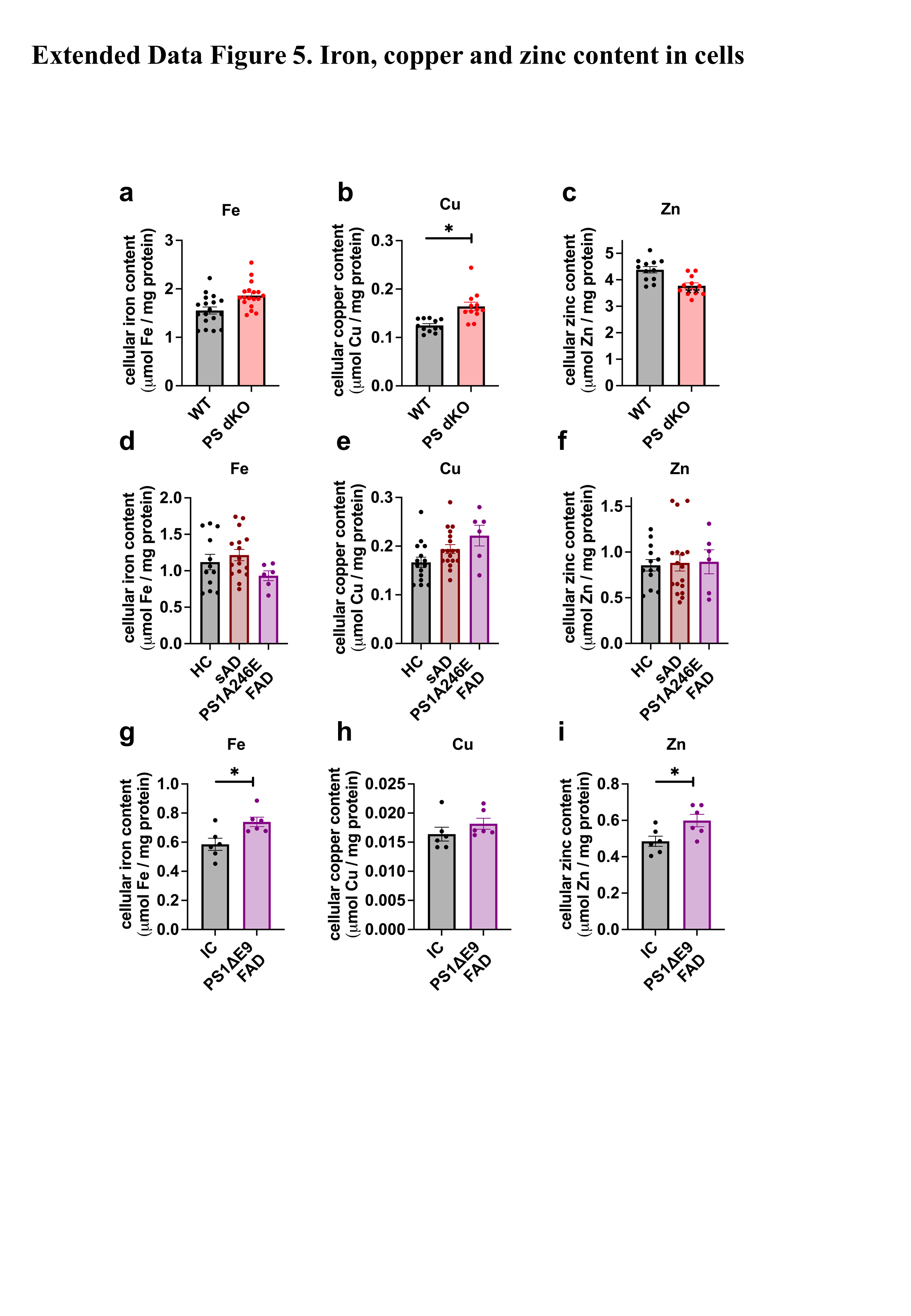

Supplement: Supplementary file 6 — Extended Data Figure 5 [file 41418_2022_1003_MOESM6_ESM.png]

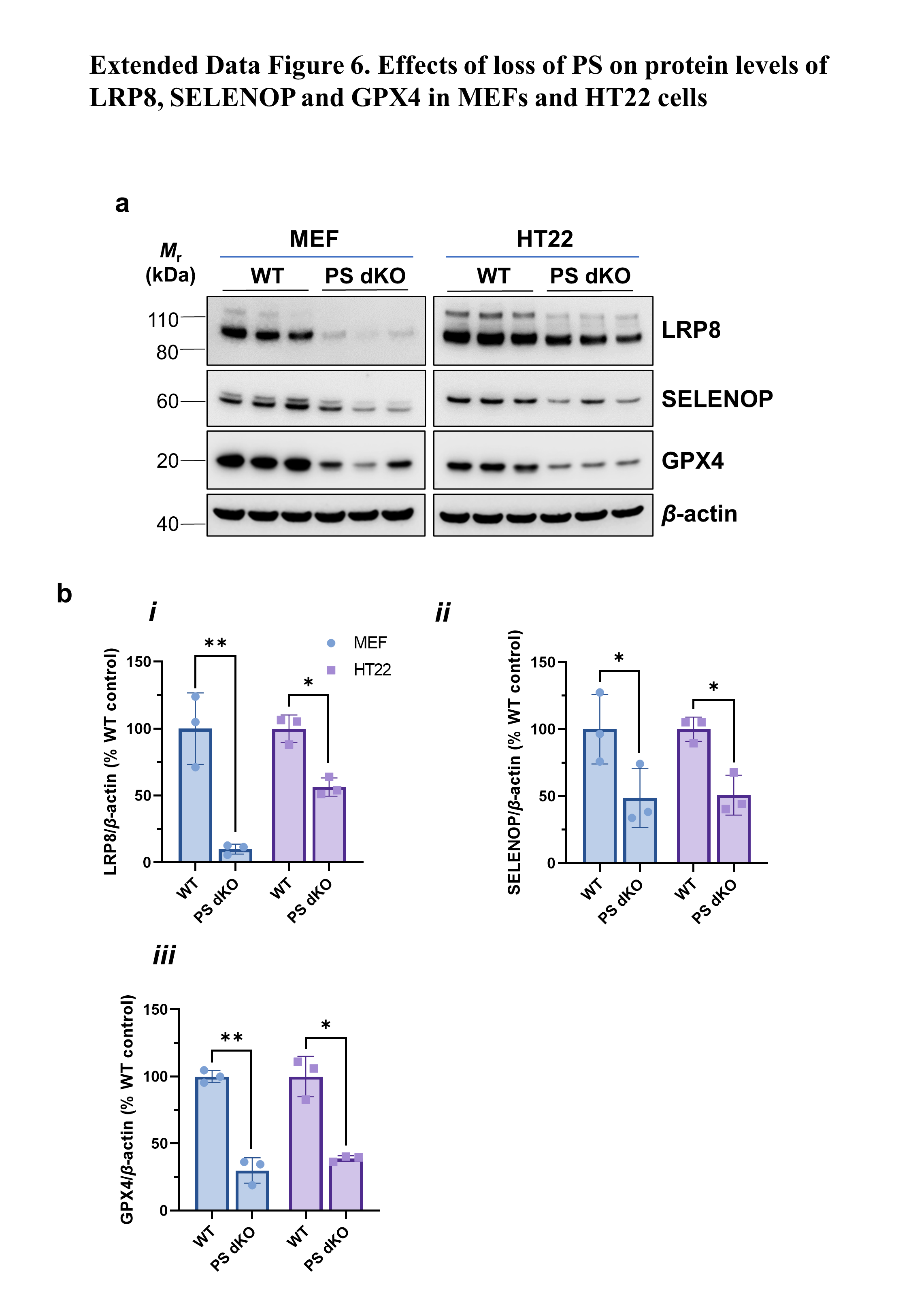

Supplement: Supplementary file 7 — Extended Data Figure 6 [file 41418_2022_1003_MOESM7_ESM.png]

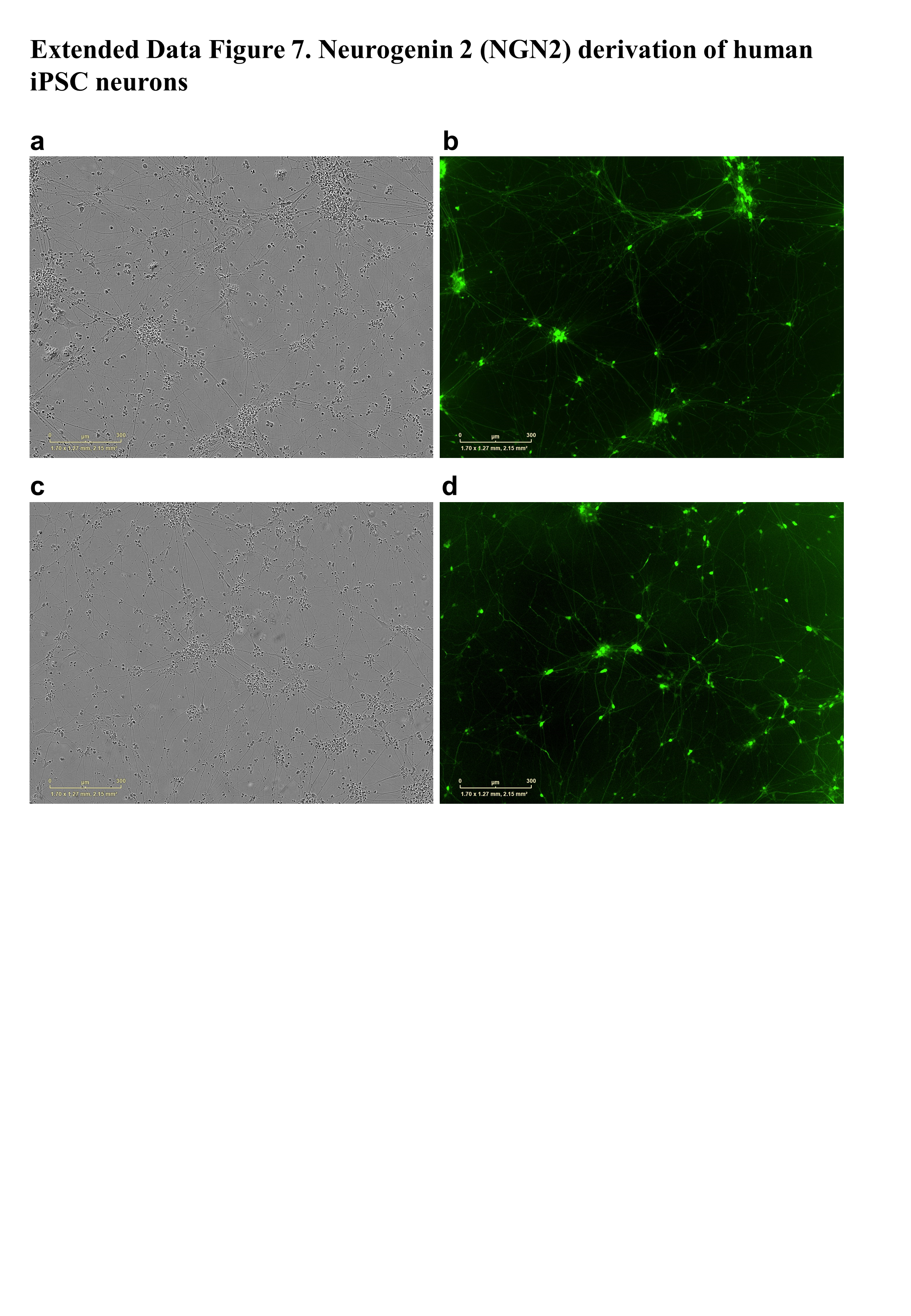

Supplement: Supplementary file 8 — Extended Data Figure 7 [file 41418_2022_1003_MOESM8_ESM.png]

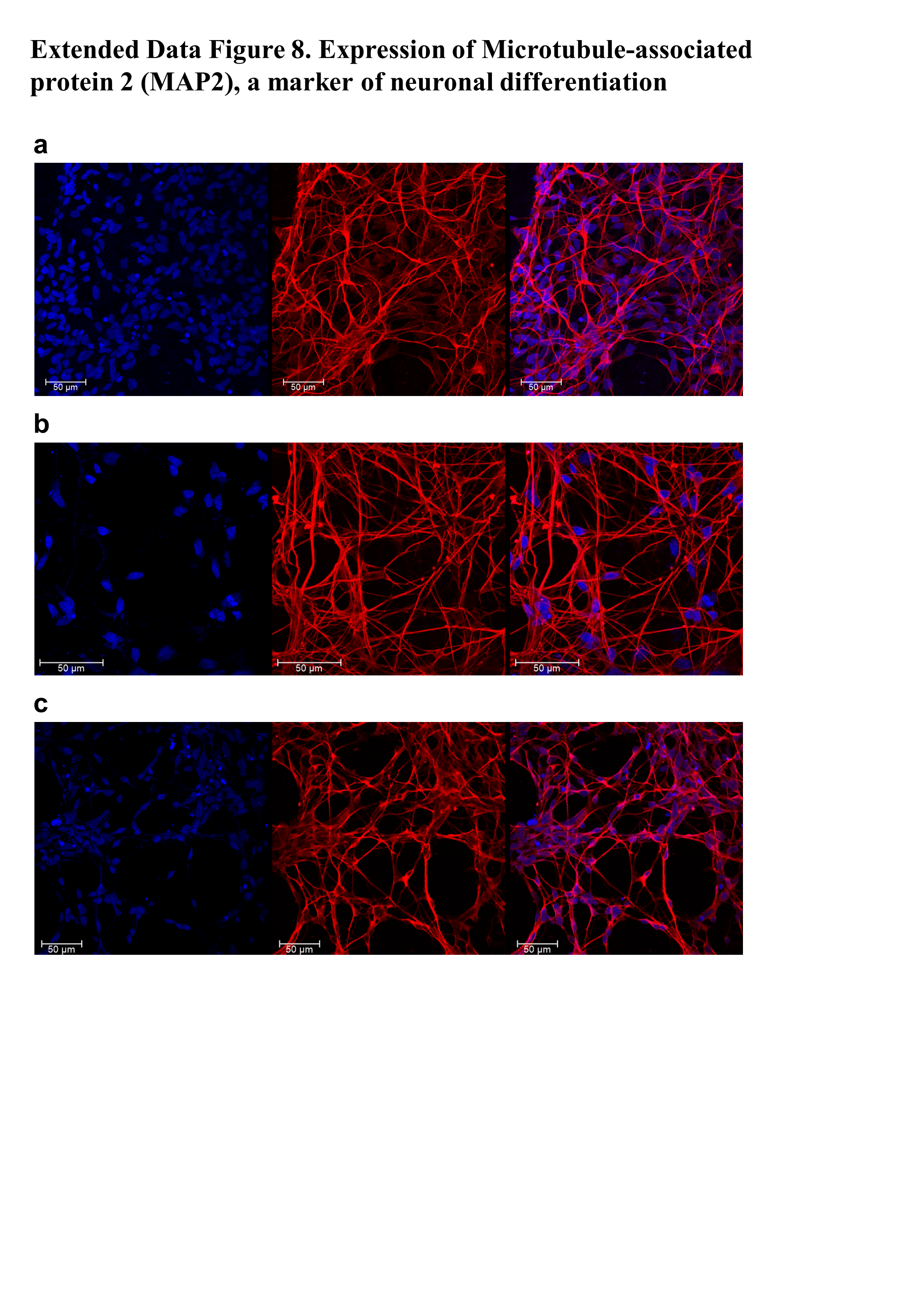

Supplement: Supplementary file 9 — Extended Data Figure 8 [file 41418_2022_1003_MOESM9_ESM.png]
