# Supplementary material for: Selective ferroptosis vulnerability due to familial Alzheimer’s disease presenilin mutations
Source: Cell Death Differ. 2022 Apr 21;29(11):2123–36. doi: 10.1038/s41418-022-01003-1 (PMC9613996; doi:10.1038/s41418-022-01003-1)
Supplement: Supplementary file 10 — Supplemental Material - Original Blots [file 41418_2022_1003_MOESM10_ESM.pptx]

## Slide 1
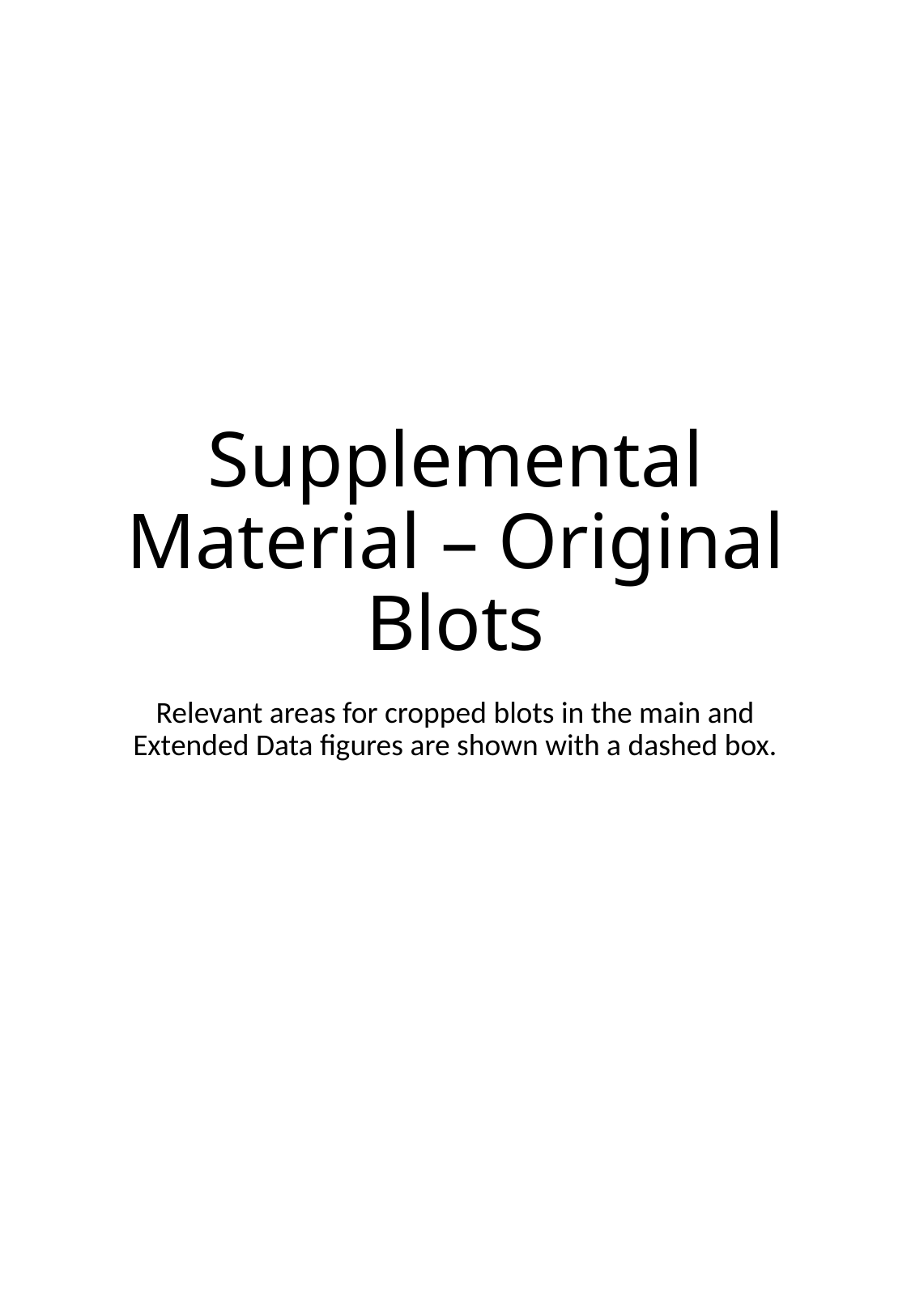

# Supplemental Material – Original Blots
Relevant areas for cropped blots in the main and Extended Data figures are shown with a dashed box.

## Slide 2
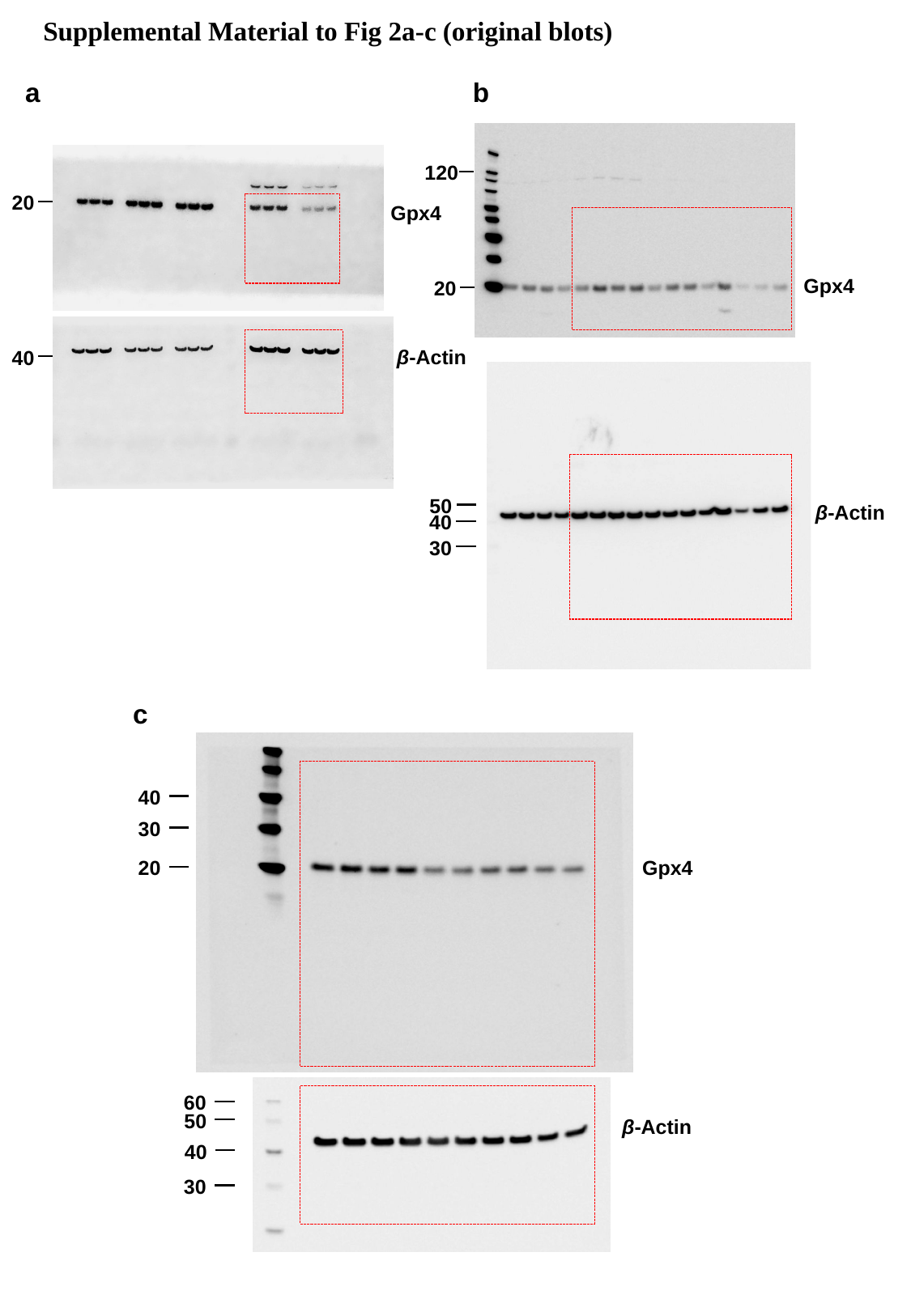

Supplemental Material to Fig 2a-c (original blots)
a
b
120
20
Gpx4
Gpx4
20
β-Actin
40
50
β-Actin
40
30
c
40
30
20
Gpx4
60
50
β-Actin
40
30

## Slide 3
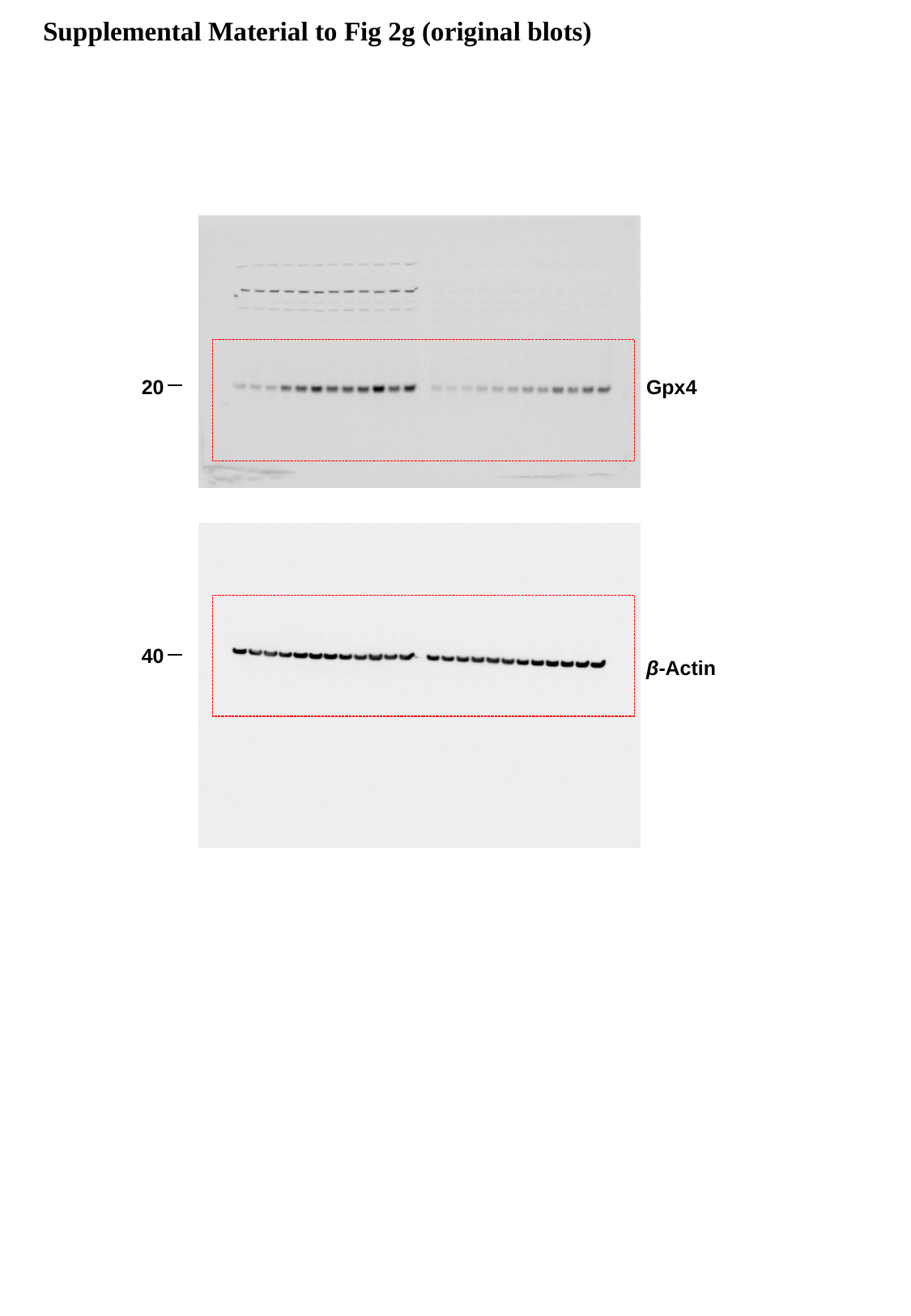

Supplemental Material to Fig 2g (original blots)
20
Gpx4
40
β-Actin

## Slide 4
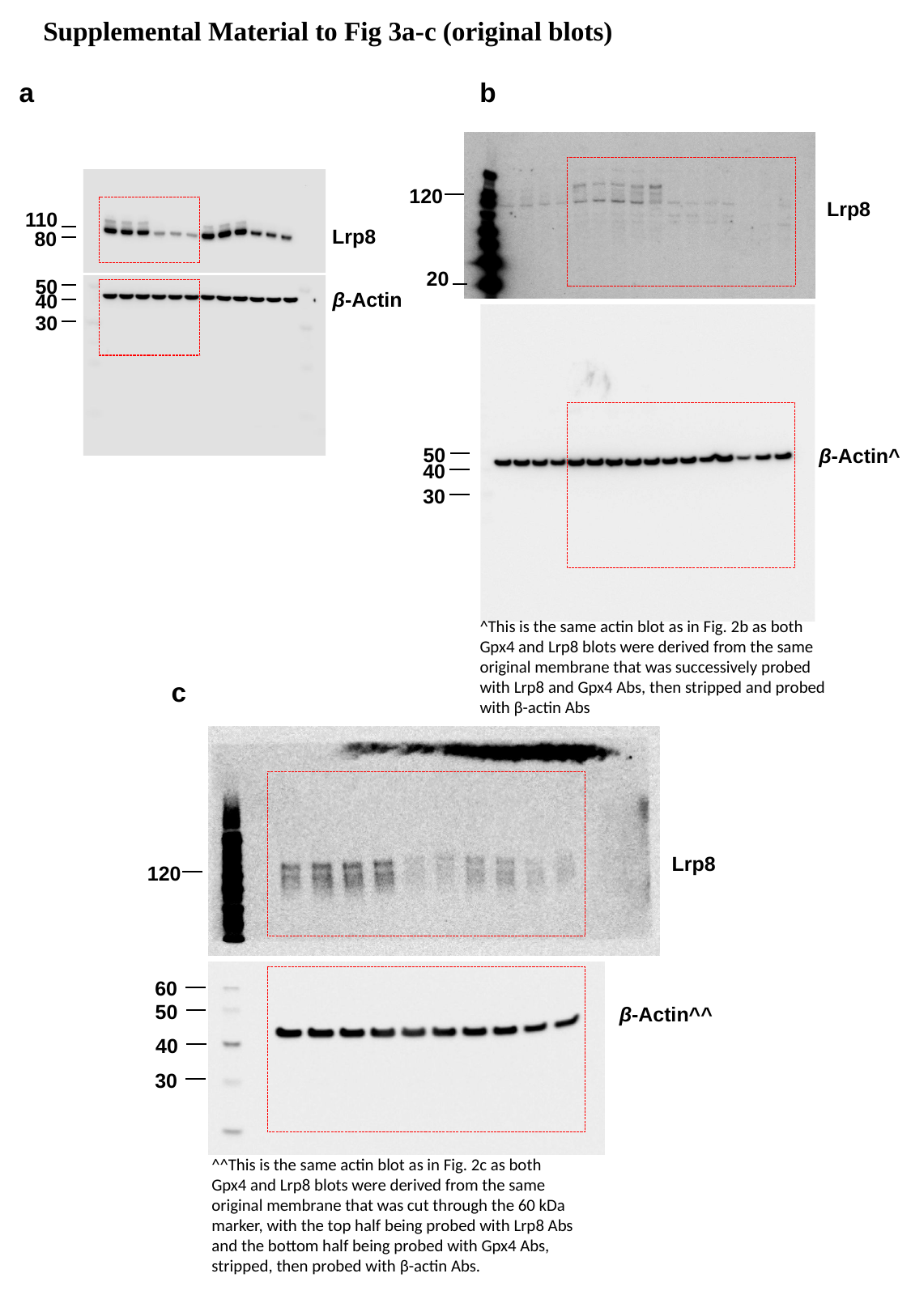

Supplemental Material to Fig 3a-c (original blots)
a
b
120
Lrp8
110
Lrp8
80
20
50
β-Actin
40
30
50
β-Actin^
40
30
^This is the same actin blot as in Fig. 2b as both Gpx4 and Lrp8 blots were derived from the same original membrane that was successively probed with Lrp8 and Gpx4 Abs, then stripped and probed with β-actin Abs
c
Lrp8
120
60
50
β-Actin^^
40
30
^^This is the same actin blot as in Fig. 2c as both Gpx4 and Lrp8 blots were derived from the same original membrane that was cut through the 60 kDa marker, with the top half being probed with Lrp8 Abs and the bottom half being probed with Gpx4 Abs, stripped, then probed with β-actin Abs.

## Slide 5
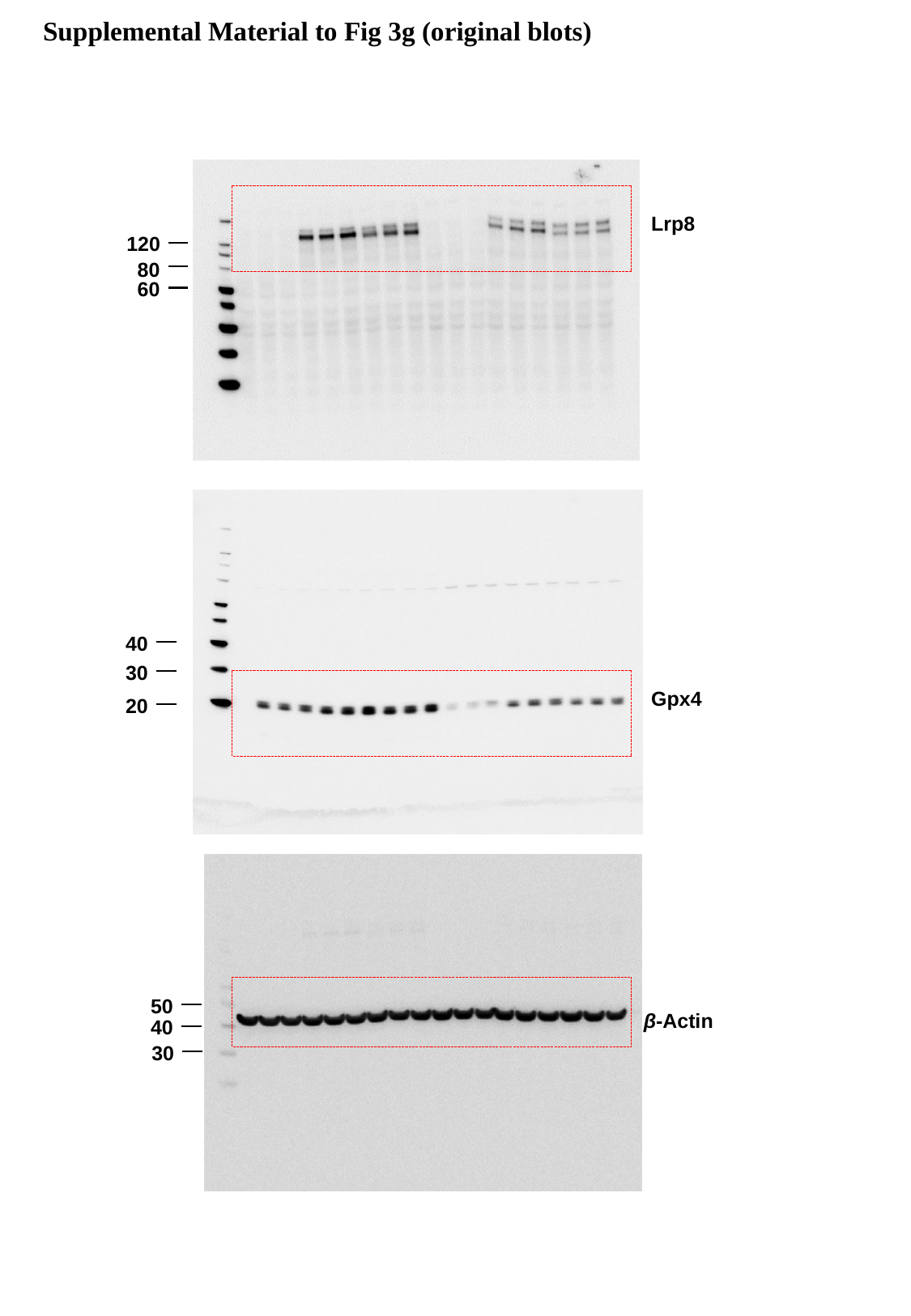

Supplemental Material to Fig 3g (original blots)
Lrp8
120
80
60
40
30
Gpx4
20
50
β-Actin
40
30

## Slide 6
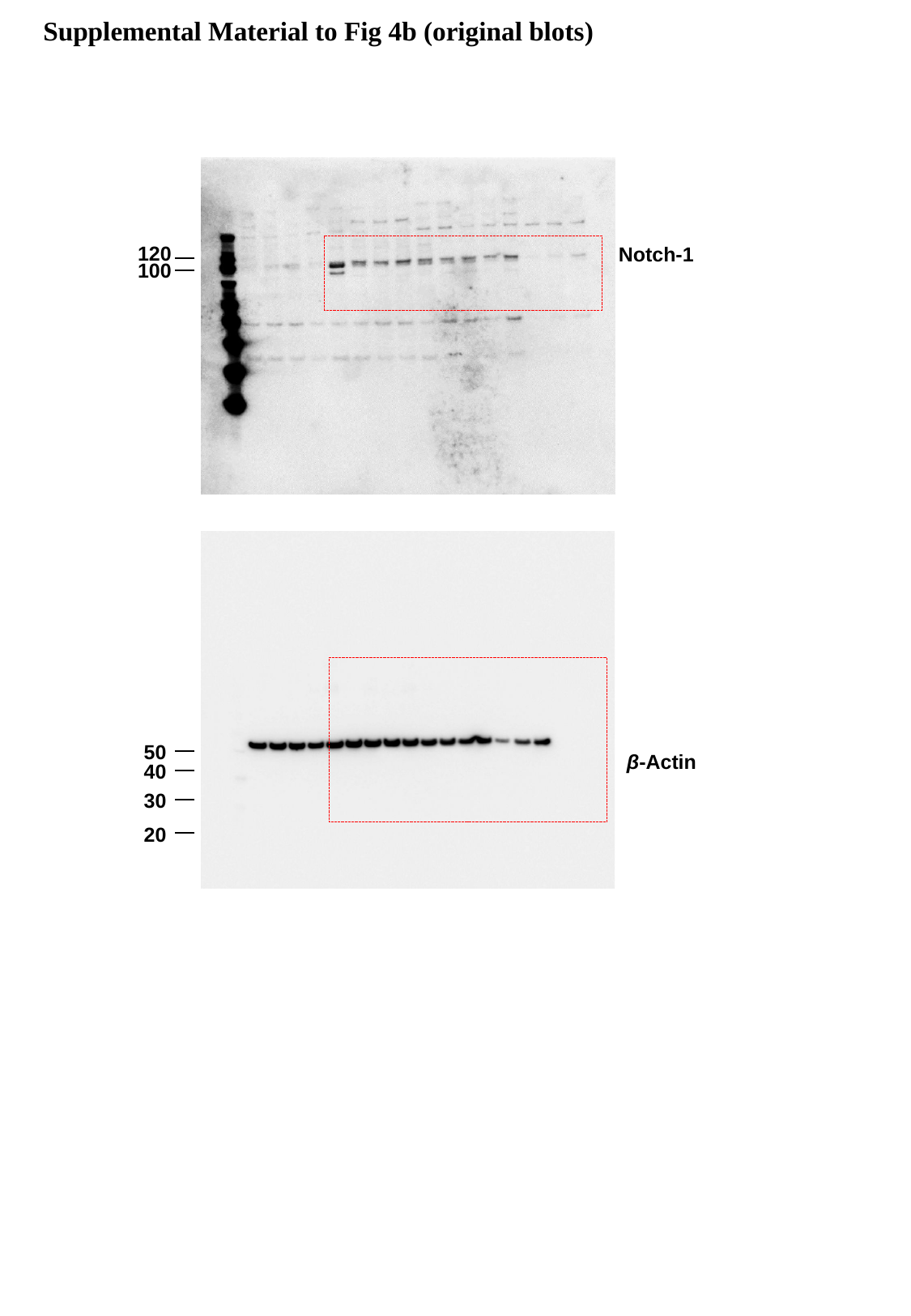

Supplemental Material to Fig 4b (original blots)
120
Notch-1
100
50
β-Actin
40
30
20

## Slide 7
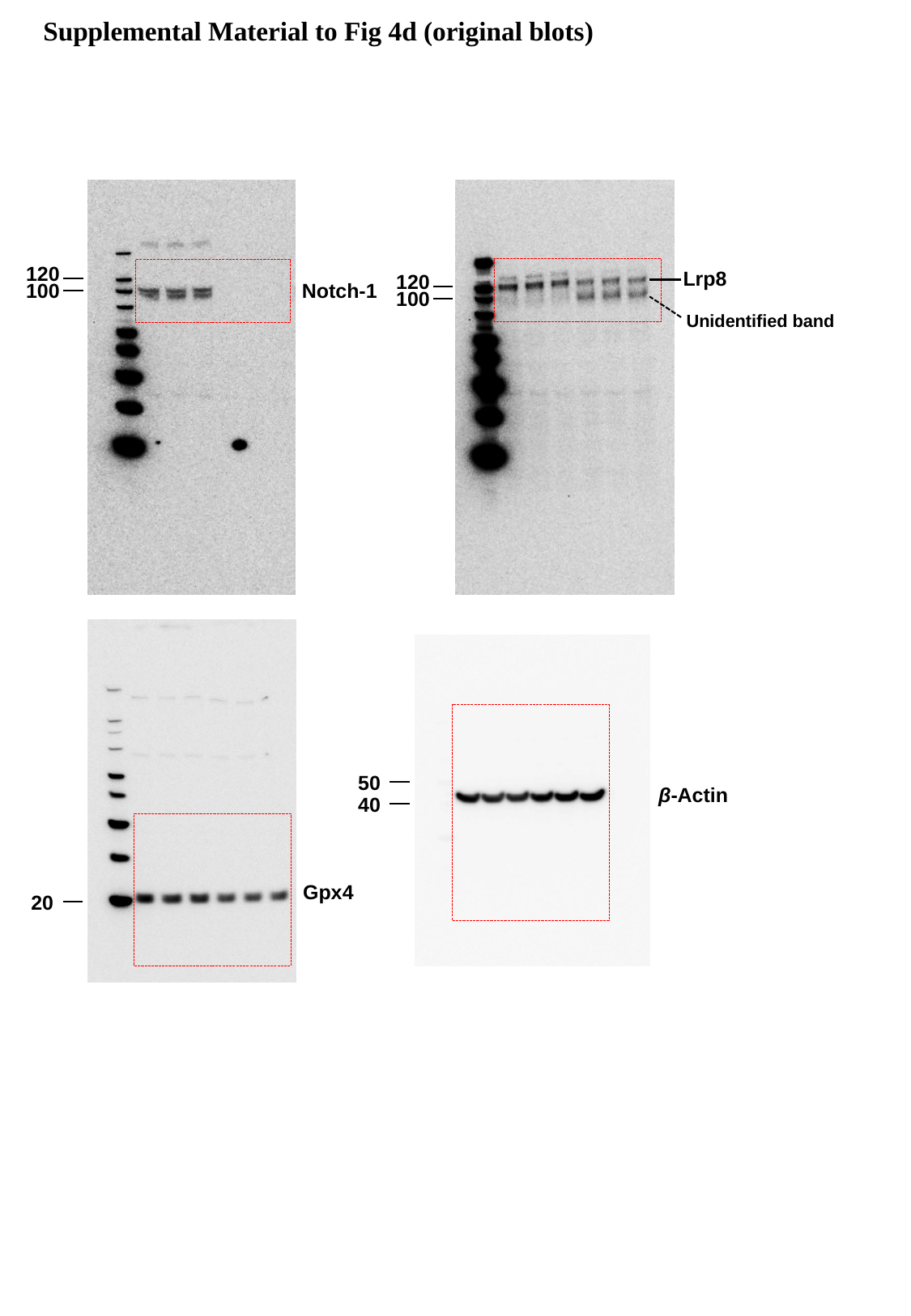

Supplemental Material to Fig 4d (original blots)
120
Lrp8
120
100
Notch-1
100
Unidentified band
50
β-Actin
40
Gpx4
20

## Slide 8
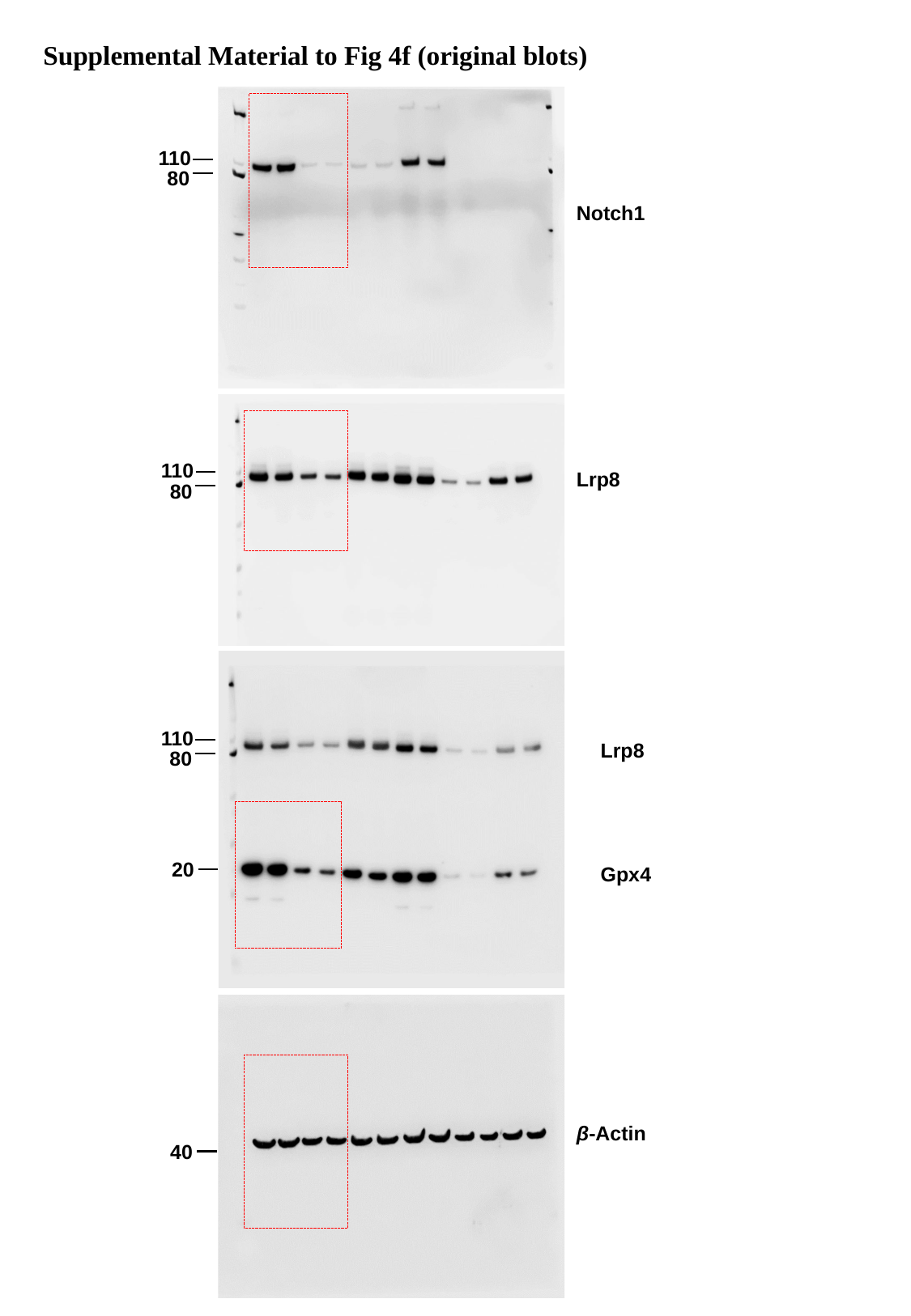

Supplemental Material to Fig 4f (original blots)
110
80
Notch1
110
Lrp8
80
110
Lrp8
80
20
Gpx4
β-Actin
40

## Slide 9
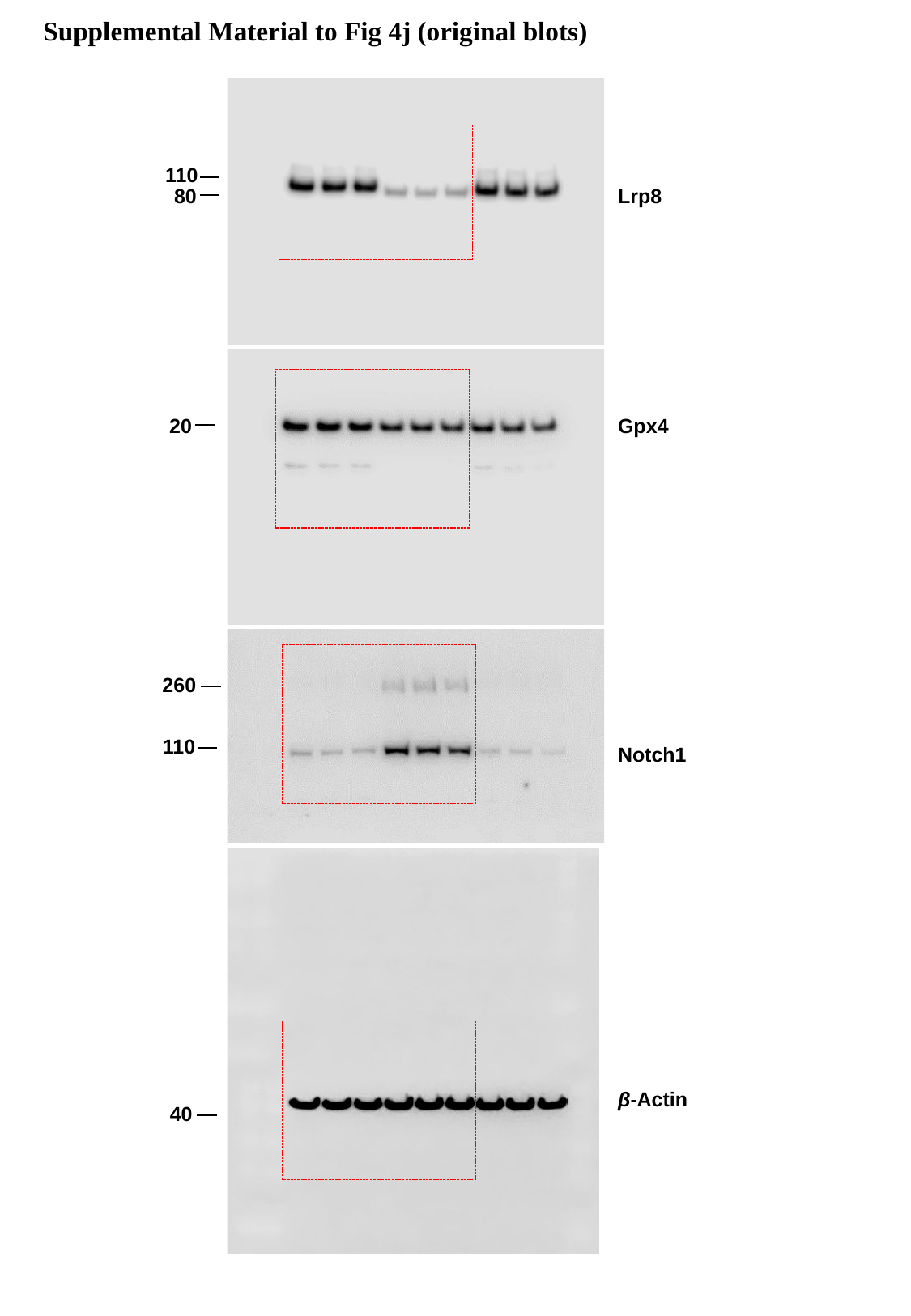

Supplemental Material to Fig 4j (original blots)
110
80
Lrp8
Gpx4
20
260
110
Notch1
β-Actin
40

## Slide 10
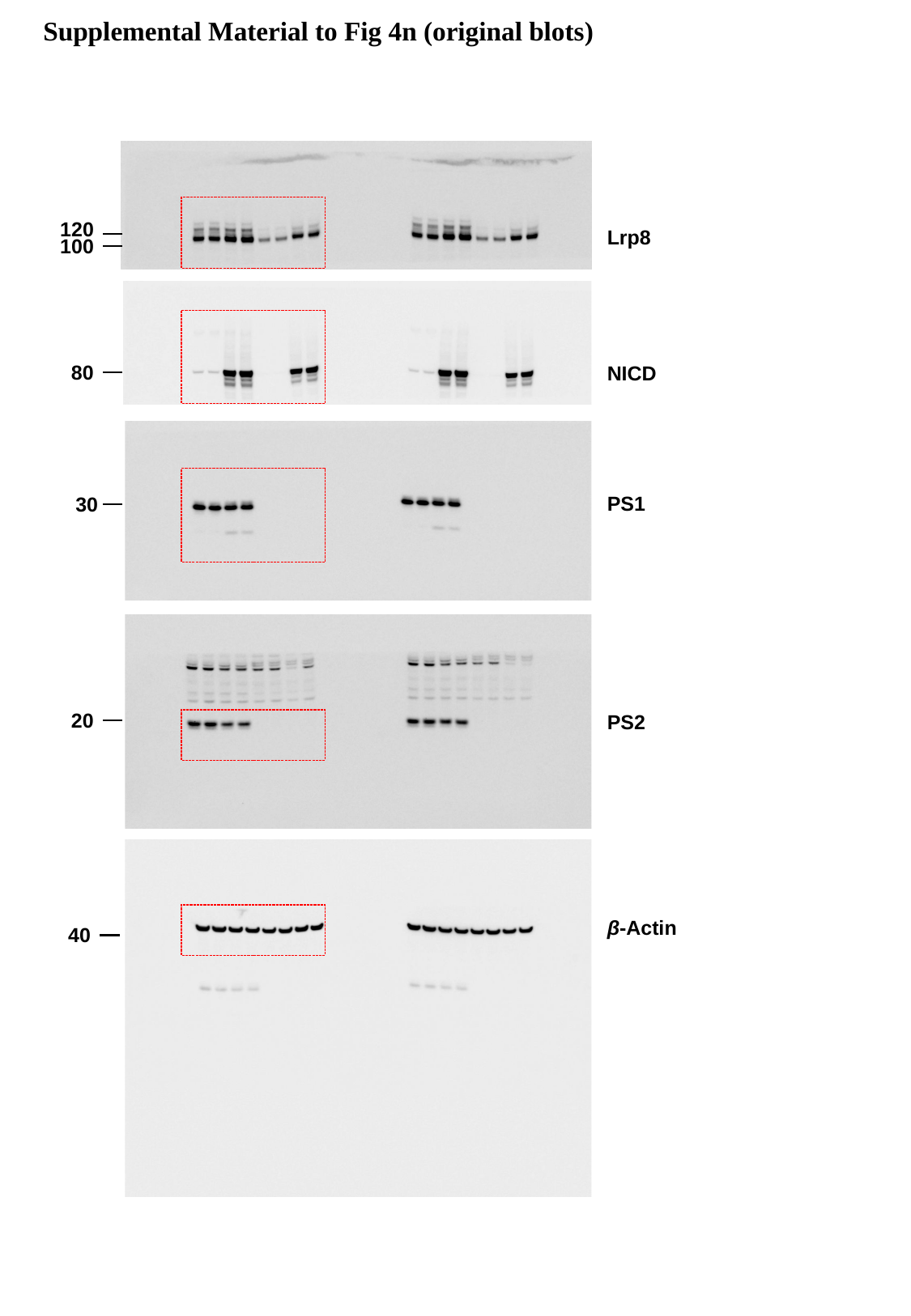

Supplemental Material to Fig 4n (original blots)
120
Lrp8
100
80
NICD
PS1
30
20
PS2
β-Actin
40

## Slide 11
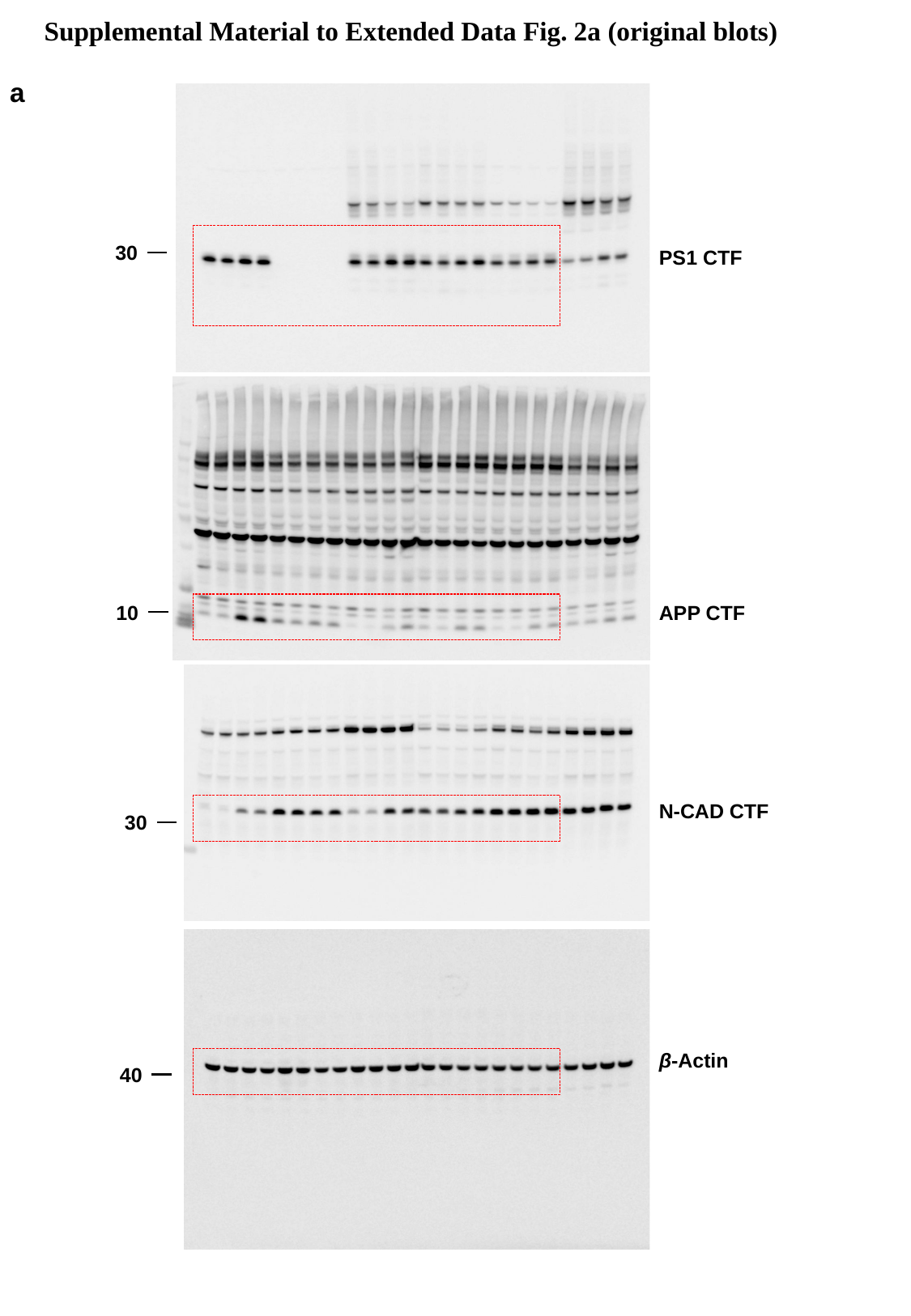

Supplemental Material to Extended Data Fig. 2a (original blots)
a
30
PS1 CTF
10
APP CTF
N-CAD CTF
30
β-Actin
40

## Slide 12
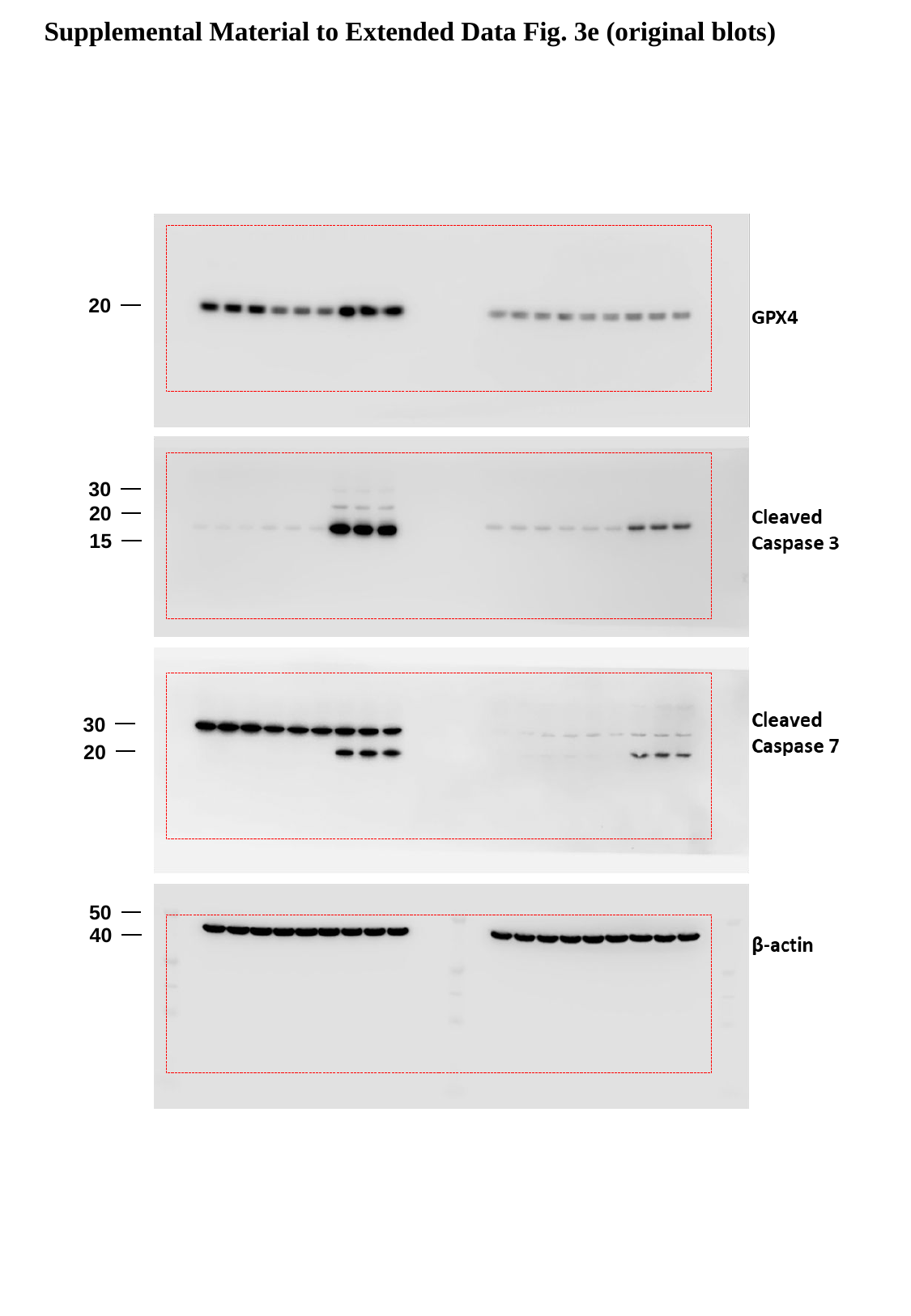

Supplemental Material to Extended Data Fig. 3e (original blots)
20
30
20
15
30
20
50
40

## Slide 13
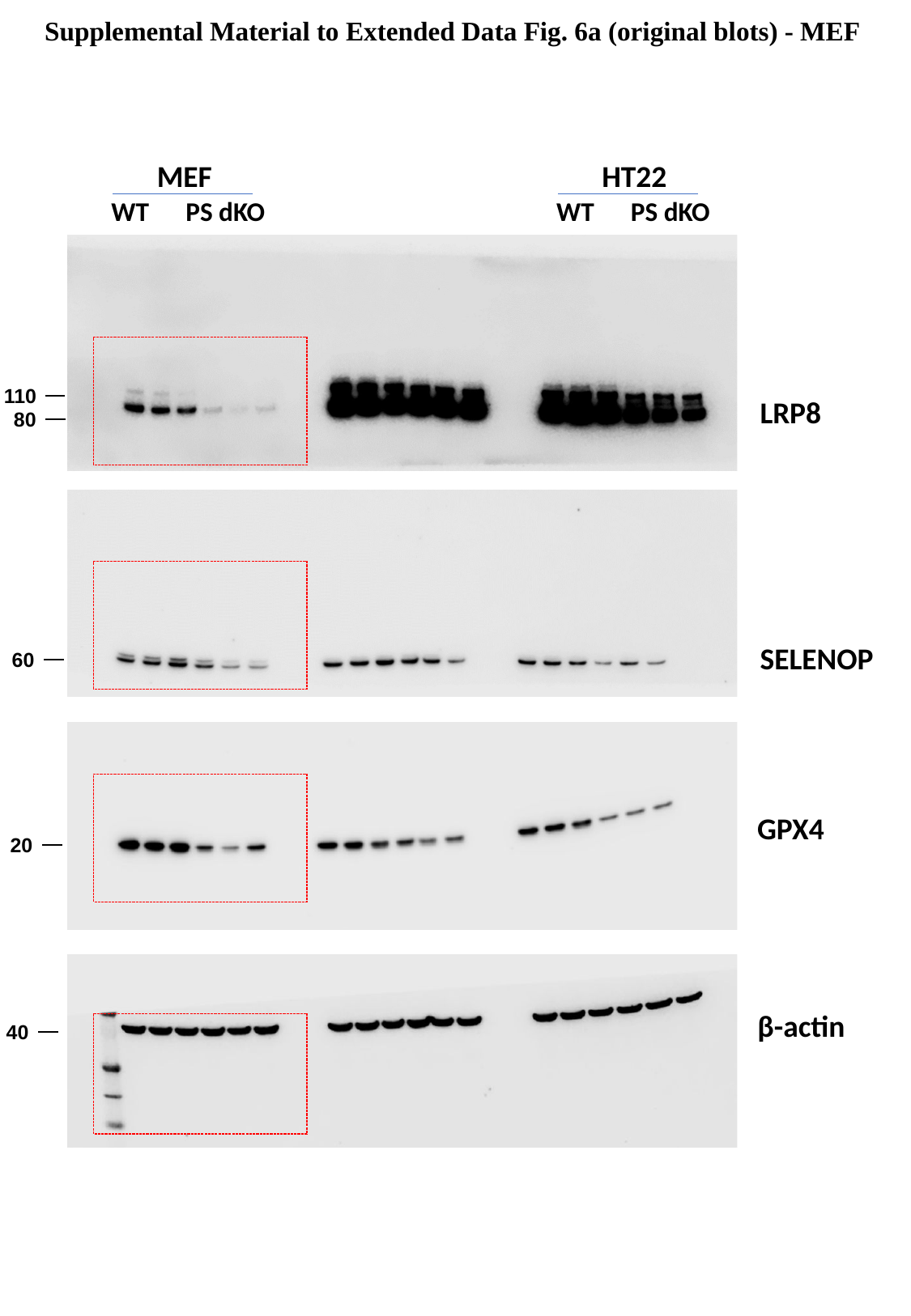

Supplemental Material to Extended Data Fig. 6a (original blots) - MEF
MEF
HT22
WT
WT
PS dKO
PS dKO
110
LRP8
80
SELENOP
60
GPX4
20
β-actin
40

## Slide 14
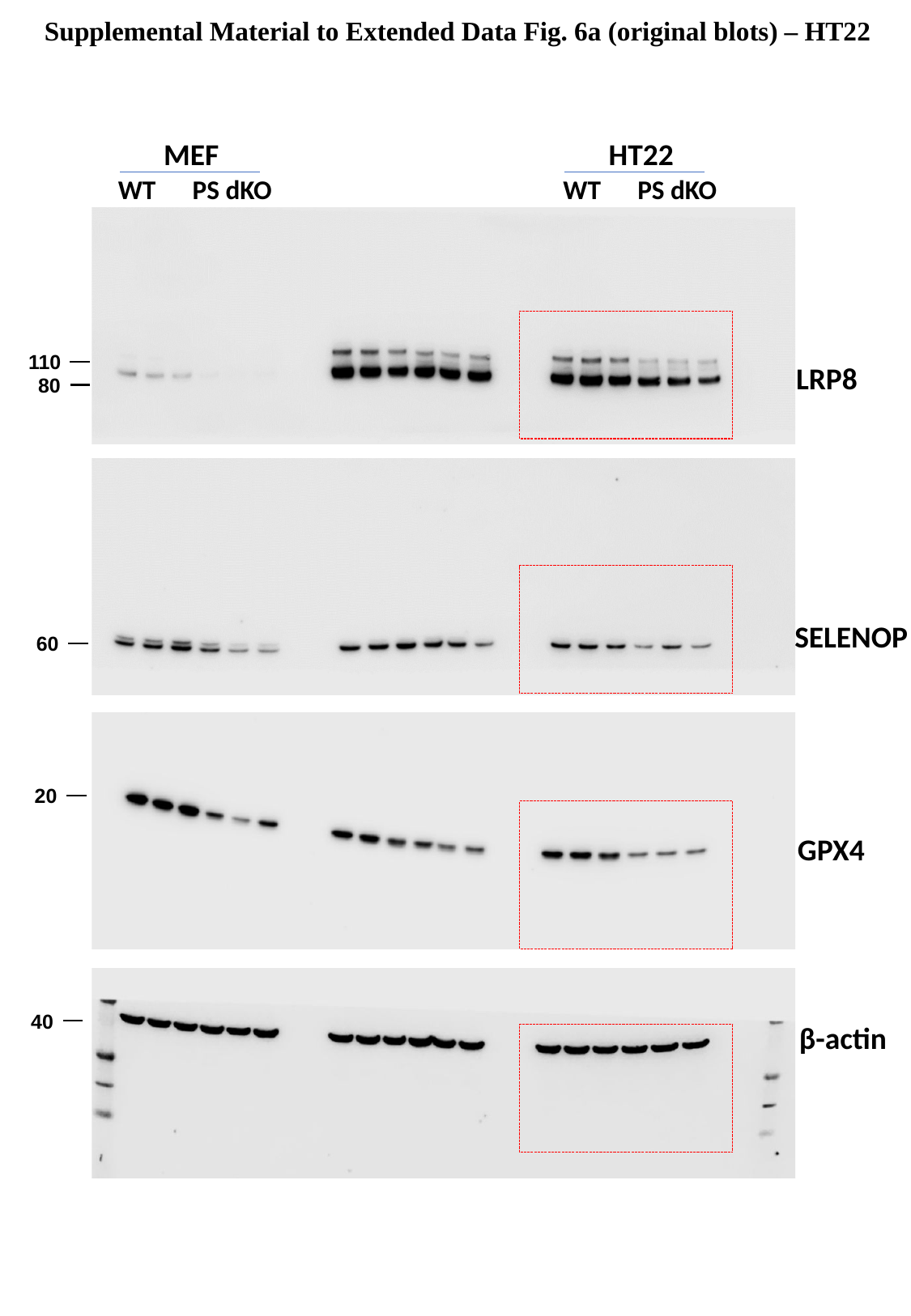

Supplemental Material to Extended Data Fig. 6a (original blots) – HT22
MEF
HT22
WT
WT
PS dKO
PS dKO
110
LRP8
80
SELENOP
60
20
GPX4
40
β-actin
